# Dark-induced senescence of barley leaves involves activation of plastid transglutaminases

**DOI:** 10.1007/s00726-014-1912-y

**Published:** 2015-01-13

**Authors:** E. Sobieszczuk-Nowicka, A. Zmienko, A. Samelak-Czajka, M. Łuczak, M. Pietrowska-Borek, R. Iorio, S. Del Duca, M. Figlerowicz, J. Legocka

**Affiliations:** 1 Department of Plant Physiology, Faculty of Biology, Adam Mickiewicz University of Poznań, ul. Umultowska 89, 61-614 Poznań, Poland; 2 Institute of Bioorganic Chemistry, Polish Academy of Sciences, ul. Noskowskiego 12/14, 61-704 Poznań, Poland; 3 Institute of Computing Science, Poznań University of Technology, ul. Piotrowo 2, 60-965 Poznan, Poland; 4 Department of Plant Physiology, Poznań University of Life Sciences, ul. Wołyńska 35, 60-637 Poznań, Poland; 5 Department of Biological, Earth and Environmental Sciences, University of Bologna, via Irnerio 42, 40126 Bologna, Italy; 6 Department of Biochemistry and Biotechnology, Poznań University of Life Sciences, ul. Wołyńska 35, 60-637 Poznań, Poland

**Keywords:** Chloroplast, Leaf, *Hv*-*Png1*-*like* gene, Polyamines, Senescence, Transglutaminases

## Abstract

Transglutaminases (E.C. 2.3.2.13) catalyze the post-translational modification of proteins by establishing ε-(γ-glutamyl) lysine isopeptide bonds and by the covalent conjugation of polyamines to endo-glutamyl residues of proteins. In light of the confirmed role of transglutaminases in animal cell apoptosis and only limited information on the role of these enzymes in plant senescence, we decided to investigate the activity of chloroplast transglutaminases (ChlTGases) and the fate of chloroplast-associated polyamines in *Hordeum vulgare* L. ‘Nagrad’ leaves, where the senescence process was induced by darkness (day 0) and continued until chloroplast degradation (day 12). Using an anti-TGase antibody, we detected on a subcellular level, the ChlTGases that were associated with destacked/degraded thylakoid membranes, and beginning on day 5, were also found in the stroma. Colorimetric and radiometric assays revealed during senescence an increase in ChlTGases enzymatic activity. The MS/MS identification of plastid proteins conjugated with exogenous polyamines had shown that the ChlTGases are engaged in the post-translational modification of proteins involved in photosystem organization, stress response, and oxidation processes. We also computationally identified the cDNA of *Hv*-*Png1*-*like*, a barley homologue of the Arabidopsis *AtPng1* gene. Its mRNA level was raised from days 3 to 10, indicating that transcriptional regulation controls the activity of barley ChlTGases. Together, the presented results deepen our knowledge of the mechanisms of the events happened in dark-induced senescence of barley leaves that might be activation of plastid transglutaminases.

**Electronic supplementary material:**

The online version of this article (doi:10.1007/s00726-014-1912-y) contains supplementary material, which is available to authorized users.

## Introduction

Transglutaminases (TGases, E.C. 2.3.2.13) are intracellular and extracellular enzymes that catalyze the post-translational modification of proteins by establishing ε-(γ-glutamyl) lysine isopeptide bonds and the covalent conjugation of polyamines to endo-glutamyl residues of protein substrates (Lorand and Graham 2003; Serafini-Fracassini and Del Duca 2008; Serafini-Fracassini et al. 2009). The few plant TGases sequenced to date have little sequence homology with the best-known animal enzymes, except for the catalytic triad; however, these TGases may share structural homology (Beninati et al. 2013; Della Mea et al. 2004a; Villalobos et al. 2004). Plant TGases, similar to their animal counterparts, are calcium-dependent, possess the ability to produce γ-glutamyl polyamine derivatives, and are also recognized by animal TGase antibodies (Beninati et al. 2013; Sobieszczuk-Nowicka et al. 2007, 2008, 2009). Plant TGases are involved in the growth and differentiation processes and are related to fertilization, greening, pollen germination, abiotic and biotic stresses (Del Duca et al. 2007; Della Mea et al. 2007; Dondini et al. 2001; Iorio et al. 2008; Serafini-Fracassini et al. 2002; Sobieszczuk-Nowicka et al. 2007, 2008).

TGase activity has been detected in algae as well as in angiosperms in various organs and subcellular compartments, and a role of chloroplast transglutaminases (ChlTGases) in the dynamic regulation of chloroplasts structure has been especially well documented. ChlTGases have been found mainly via immunodetection in maize callus and cucumber cotyledons, as well as in the leaves of barley, potato, tomato, *Arabidopsis thaliana*, *Helianthus tuberosus*, and *Nicotiana tabacum* (Serafini-Fracassini and Del Duca 2008). Villalobos et al. (2004) demonstrated that ChlTGases may be involved in the regulation of the ratio of grana thylakoids to stroma thylakoids. The architecture of the thylakoids is a major factor affecting the functionality and efficiency of the photosynthetic apparatus. Ioannidis et al. (2009) demonstrated that the remodeling of the grana may be achieved by the over-expression of a ChlTGase gene and suggested that this enzyme plays an important functional role in the formation of grana stacks. Available data strongly suggest that the mechanisms of thylakoid structure regulation involve polyamine binding to chloroplast proteins, mediated by ChlTGases (Sobieszczuk-Nowicka and Legocka 2014). The main representatives of polyamines in plant cells are putrescine (PU), spermidine (SD), and spermine (SM) (Takahashi and Kakehi 2010). An increase in the ChlTGase activity in the transformed tobacco plants resulted in the enhanced incorporation of polyamines into thylakoid proteins and in increased thylakoid oppression (Ioannidis et al. 2009). It has been suggested that ChlTGases may bind polyamines to antenna proteins of light-harvesting chlorophyll *a*/*b*-protein complex (LHCII) to proteins of chlorophyll-protein complexes (CPs) transducing excitation energy: CP24, CP26, CP29, as well as to the larger subunit of ribulose bisphosphate carboxylase-oxygenase, thereby participating in the processes of chloroplast formation, photoprotection, and the stabilization of LHCII (Del Duca et al. 1994; Dondini et al. 2003; Margosiak et al. 1990; Sobieszczuk-Nowicka et al. 2008).

Structural changes of the chloroplasts, eventually leading to chloroplasts degradation mark the first phase of a sequential process of leaf senescence, both developmental one as well as induced by stresses (Sarwat et al. 2013). Senescence involves a highly regulated series of transformations, both cytological and biochemical, involving the cessation of photosynthesis, the disintegration of chloroplasts, the breakdown of leaf proteins, the loss of chlorophyll, and the remobilization of nitrogen and carbon compounds like amino acids, to other parts of plant (Buchannan-Wollaston et al. 2003). Finally, disruption of other organelles (nucleus and mitochondrion) takes place. Significant chromatin condensation, internucleosomal fragmentation of nuclear DNA and enhanced expression of cysteine proteases in the senescing mesophyll demonstrate that leaf senescence is a genetically defined process involving the mechanisms of programmed cell death (Buchannan-Wollaston and Ainsworth 1997; Van Doorn and Yoshimoto 2010). Interestingly, the dying process of a plant cell may be reversible as long as the functions of the chloroplasts can be restored (Van Doorn and Yoshimoto 2010). Therefore, we hypothesized that activation of ChlTGases may be important for executing the tightly regulated scenario of leaf senescence. Their likely contribution is also underscored by the participation of polyamines in this process. Polyamines had an anti-senescing effect when added exogenously to detached barley leaves that were incubated in darkness or to senescing lettuce leaves. Treating senescing leaves with SD and SM inhibited the degradation of thylakoids and chlorophyll (Legocka and Zajchert 1999; Serafini-Fracassini et al. 2010). However, little information is available regarding the actual role of TGases in plant cell senescence. The few available studies have focused on cell death associated with both the hypersensitive reactions to pathogens and developmental petals senescence (Del Duca et al. 2007; Della Mea et al. 2007; Dondini et al. 2001; Serafini-Fracassini et al. 2002). At the same time, involvement of both TGases and polyamines in animal cell apoptosis is well documented (Fraij 2014; Griffin and Verderio 2000; Kikuchi et al. 2014; Lorand and Graham 2003). While their activity in growing animal cells is low, the increased TGase gene expression and protein accumulation are associated with animal cell death. A clear role for animal TGase was revealed in the formation of apoptotic bodies (Lorand and Graham 2003). Although it has been suggested that in many mammalian systems TGase is a downstream effector in the later stages of apoptosis, elevated TGase accumulation has also been connected with early apoptotic events (Fesus et al. 1989; Griffin and Verderio 2000; Piacentini and Melino 1994).

Scientific work from the leaf senescence field has focused primarily on the model plant *Arabidopsis thaliana*, secondly on the identification and regulation of leaf senescence and remarkably less is known about the mechanisms of the major events in this process. In light of the confirmed role of TGase in animal cell apoptosis and only limited information on the role of this enzyme in leaf senescence, we decided to investigate the activity of ChlTGases and the fate of chloroplast-associated polyamines in barley leaves. We chose a model process that we routinely use for senescence-related studies in which senescence in barley is induced by incubation in the dark (Jackowski 1996; Legocka and Szweykowska 1981; Żelisko and Jackowski 2004).

## Materials and methods

### Plant material

Barley (*Hordeum vulgare* L. ‘Nagrad’) seedlings were grown for 7 days on soil under controlled conditions (day/night 16/8 h, 23 °C, light intensity 150 μmol m^−2^ s^−1^, 60 % humidity) (the material for the day 0 sample was then collected). Light limitation initiated the onset of senescence and allowed the leaves to senesce in the darkness for 3, 5, 7, 10, or 12 days. Plastid isolation was performed following the procedure described previously (Sobieszczuk-Nowicka et al. 2008) by a protocol based on a differential centrifugation of leaf homogenate. The intactness and purity of the isolated plastids were evaluated by phase contrast microscopy as well as by enzymatic and immunological approaches (glutamate dehydrogenates, a mitochondrial marker enzyme, UDP-glucose pyrophosphorylase, cytosolic marker enzyme). These analyses confirmed that the samples could be reliably used for subsequent studies.

### HPLC polyamine analysis

Quantitative and qualitative analysis of polyamines was performed by HPLC method according to Marcé et al. (1995) using the Varian chromatograph. The pellet of plastids (100 mg ml^−1^) was suspended in 5 % perchloric acid, and plastid-bound polyamines were released by hydrolysis in 6 M HCl for 18 h at 110 °C and then dansylated with dansyl chloride. Dansylated polyamines were collected with toluene and, after toluene evaporation, dissolved in 800 µl acetonitrile. The sample (5–20 µl) was applied to a column of Spherisorb 5 µm ODS2^®^ (4.6 × 100 mm, Waters) with guard column C18 (Supelguard™ Discovery^®^ 2 cm × 4.0 mm, 5 µm, Supelco); flow rate 1.5 ml min^−1^. Gradient elution was performed with acetonitrile (solvent A) and water (solvent B): 0–4 min, 70 % A; 4–5 min, 70–100 % A; 5–9 min, 100 % A; 9–10 min, 100–70 % A; 10–15 min, 70 % A. The flow of dansylated, polyamines was monitored using a fluorescence detector–Prostar 363 (excitation at 252 nm, emission at 500 nm). Retention times of the different polyamines were as follows: 1.8 min for DP, 2.2 min for PU, 3.35 min for DH (diaminoheptane, internal reference), 5.46 min for SD and 6.63 min for SM.

### TGase protein identification

Immunocytochemistry and autoradiography were performed as described by Sobieszczuk-Nowicka et al. (2008). For protein identification, the anti-TGase antibody (RB-060, Ab-4, Neo-Markers™, Fremont, CA, USA) was used.

### TGase activity assays

Colorimetric assay of TGase activity. The biotin-cadaverine incorporation assay was carried out as described by Lilley et al. (1998). Endogenous TGase activity in isolated chloroplasts was measured as covalent binding of biotinylated cadaverine to endo-glutamyl residues of *N’,N*’-dimethylcasein (DMC) (10 mg/ml) used to precoat the assay microplate. The measurement of enzyme activity was preceded by overnight dialysis (10 mM Tris, pH 8.5 and 1 mM 2-mercaptoethanol) and performed with 5 mM Ca^2+^; replaced by 1 mM EDTA in the negative control.

For the radiometric assay, the measurement of enzyme activity was preceded by an overnight dialysis (10 mM Tris, pH 8.5, and 1 mM 2-mercaptoethanol) and performed with 2.5 mM Ca^2+^. The isolated plastids were assayed in the presence of [^3^H]PU or [^3^H]SD, which provided the primary amino groups used as the acyl acceptors of the glutamyl residues of the plastid proteins (Dondini et al. 2003). ​The assay was performed for 1 h at 37 °C in 100 mM Tris (pH 8.5) containing per sample 200 μg plastid proteins, 10 mM DTT, 3 μCi PU or SD (the specific activity of PU was 40 Ci mmol^−1^ and that of SD was 34.82 Ci mmol^−1^) and 200 μM unlabeled PU or SD, respectively. The final volume was adjusted to 300 µl with Tris. The reaction was halted by 5 % (w/v) TCA, which also contained 2 mM unlabeled PU or SD (Del Duca et al. 1994). The pigments were removed by anhydrous acetone. The incorporation of labeled PA was measured using a Beckman LS 6500 multipurpose scintillation counter.

### Mass spectrometry-based identification of TGase protein substrates

The [^3^H]PU- or [^3^H]SD-conjugated plastid proteins were labeled and imaged as described by Sobieszczuk-Nowicka et al. (2008). The protein bands were manually excised from the gels, transferred to Eppendorf tubes, destained, washed, and in-gel digested as follows. The gel pieces were rinsed twice in 100 µl washing buffer [50 mM NH_4_HCO_3_/100 % acetonitrile (vol. 1:1)] for 15 min, dehydrated in 100 % ACN, and then reduced with 10 mM DTT in 50 mM NH_4_HCO_3_ at 56 °C for 45 min and alkylated with 55 mM IAA in 50 mM NH_4_HCO_3_ at room temperature for 30 min in the dark. After the dehydration and drying, the gel pieces were rehydrated by the addition of 10 µl digestion buffer [25 mM ammonium bicarbonate and 0.2 µg sequencing-grade trypsin (Promega)]. The digestion was performed overnight at 37 °C. The peptides were extracted with 10 % ACN. The digested proteins were identified using a MALDI–TOF/TOF mass spectrometer. The acquisition of the MALDI spectra used a ultrafleXtreme mass spectrometer (Bruker Daltonics, Germany) that was operated in reflector mode, using delayed ion extraction. The positively charged ions in the 820–3,500 *m/z* range were analyzed. A total of 0.5 µl of the sample was co-crystallized with the CHCA matrix and spotted directly on the MALDI AnchorChip target (Bruker Daltonics). To validate the data, an external calibration was performed with a standard mixture of peptides. Flex control v. 3.3 software was used for the acquisition of spectra, and all further data processing was carried out using Flex analysis v. 3.3 software. The spectrometric analysis was performed in an automatic dependent mode, using Bio Tools 3.2. A maximum of ten precursor ions per sample were chosen for the MS/MS analysis. The monoisotopic peptide masses were assigned and used for database research. The protein database searches, using the combined PMF and MS/MS datasets, were performed using Bio Tools 3.2 software (Bruker). The proteins were identified using the Mascot (Matrix Science, London, UK) program against the SwissProt and NCBI databases. The protein search was performed using the following search parameters: mass tolerance ±0.5 Da, one allowed missed cleavage, cysteine treated with iodoacetamide to form carbamidomethyl-cysteine and methionine in the oxidized form.

### Identification of polyamine derivatives

The polyamine derivatives that covalently bound to the proteins were identified in the TCA-insoluble fractions obtained from the TGase radiolabeled assay, as described by Beninati et al. (2013), except that the Jasco HPLC instrument was used for chromatographic separation.

### Real-time RT-PCR

The frozen barley shoot samples from four biological replicates of the dark-induced senescence experiment (independent cultivations) were used for time course analysis. For each time point, RNA samples were prepared from a pool of ~30 plants per replicate. The RNA isolation, DNase digestion, quality analysis, reverse transcription, and qPCR assays were performed as described by Kulik et al. (2012) with the following modifications. The Rotor-GeneQ (Qiagen) qPCR system was used. The qPCR reaction was performed for 40 cycles. The working concentration of the cDNA samples was adjusted so that the amplicons would lie within the non-inhibitory concentration range defined by the 6-point standard curve which was prepared by serial dilution of a pool of equal concentrations of each cDNA, according to the guidelines presented by Gallup and Ackermann (2008). The primer sequences for the *HvPng1*-*like* expression assay were designed using Primer-BLAST (NCBI): forward (5′-CAGGCGGAAGAAGAGGCACT-3′), and reverse (5′-ACTTCAGAACCTGGCGCATGTA-3′). Each sample was run in three technical replicates. The expected size and uniformity of the product (117 bp) were verified by a melting curve analysis (60–95 °C range with 0.5 °C steps) following each qPCR assay and by agarose gel electrophoresis.

### Statistical analysis

The differences in the measured parameters were analyzed for statistical significance using one-way analysis of variance (ANOVA) and Tukey–Kramer Multiple Comparison Test. Means were considered as significantly different at *p* value <0.01.

The statistical analysis was performed using Standard Method STATISTICA software (Stat Soft Inc., Tulsa, OK, USA). For the analysis of the real-time RT-PCR data, Ct values were calculated with the Rotor-GeneQ software and the data for technical replicates were averaged. Relative gene expression was estimated based on data from four biological replicates, using the relative expression software tool (REST 2009 V2.0.13, QIAGEN, Hilden, Germany) which is a dedicated standalone software for estimation of gene expression and statistical analysis. REST 2009 V2.0.13 corrects data for exact PCR efficiencies and addresses issues surrounding the measurement of uncertainty in expression ratios by introducing randomization and bootstrapping techniques (Pfaffl et al. 2002). The expression ratios were tested for significance (*p* < 0.05) by a Pair Wise Fixed Reallocation Randomisation Test implemented in REST with 10,000 iterations.

### Bioinformatic analysis

The sequences of plant proteins that were previously reported to possess TGase activity were used for blastp and tblastn searches against the NCBI/GenBank and MaizeGDB databases, and the taxonomy term-based specific filters were applied when necessary (Altschul et al. 1997, 2005). The UniGene sequences were identified in the NCBI/UniGene database and assembled into contigs using a standalone version of the CAP3 assembler (Huang and Madan 1999). The NCBI/CDD database was searched to identify protein domains (Marchler-Bauer et al. 2011). Multiple sequence alignments were performed using the ClustalW and Multalign web-based services (Corpet 1988; Larkin et al. 2007). An Affymetrix probeset corresponding to the *HvPng1*-*like* cDNA sequence (Contig18871_at) was identified in the PLEXdb database. The expression profiles of *HvPng1*-*like* were analyzed according to the publically available results of microarray experiments via the Genevestigator database (Hruz et al. 2008) and PLEXdb database (Druka et al. 2006).

## Results

### Detection of ChlTGases in green and senescing barley leaves

To verify whether the capacity of plant to control senescence might be linked to ChlTGase-mediated changes in chloroplast architecture, we first aimed to detect ChlTGases in plastids.

**Fig. 1:**
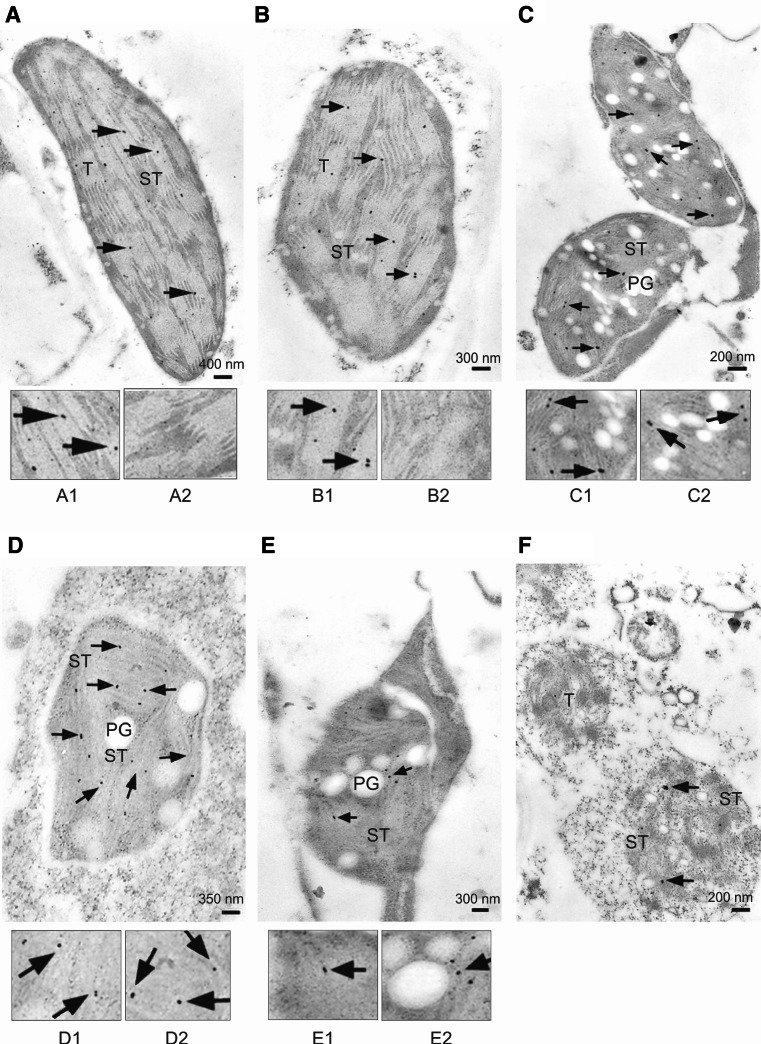
Immunolocalization of barley ChlTGase during senescence-associated leaf chloroplast degradation. **a** *A1* and *A2* ultrastructure of green leaf chloroplast in the control (day 0), **b** *B1* and *B2* and, **c** *C1* and *C2*, dark-induced initiation of thylakoid destacking during senescence (days 3 and 5, respectively), **d** *D1* and *D2*, complete thylakoid destacking, visible as parallel destacked membranes, accompanied by an increased number of plastoglobuli (day 7), **e** *E1* and *E2* disappearance of thylakoid membrane with the concomitant appearance of large plastoglobuli (day 10), **f** total gerontoplast degradation (day 12). Enlargement of thylakoid and stroma areas are presented in *A1*–*E1* and in *A2*–*E2*, respectively. The gold particles (*arrowheads*) indicate TGase localization. *T* thylakoid, *ST* stroma, *PG* plastoglobuli

We observed changes in the suborganellar localization of the ChlTGases during the chloroplast-to-gerontoplast transformation and during the final degradation of the gerontoplast that was associated with the senescence process (Fig. 1). The primary changes in the ultrastructure of the chloroplasts during senescence involved thylakoid membrane destacking and the appearance of plastoglobuli (days 3–5, Fig. 1b, c). Over time, parallel destacked membranes were observed and eventually disappeared by the end of the process, which was accompanied by an increase in the number and size of the plastoglobuli (days 7–12, Fig. 1d–f). Gold labeling revealed the presence of ChlTGases at all stages of dark-induced chloroplast degradation. Initially, the enzyme specifically localized to the thylakoids, where it persisted until their disassembly (days 0–10, Fig. 1a–e, 1A1–E1), but as the degradation advanced, the ChlTGases were detected in the stroma as well (days 5–12, Fig. 1c–f, 1C2–E2).

### Changes in the level of plastid-associated polyamines during senescence of barley leaves

**Fig. 2:**
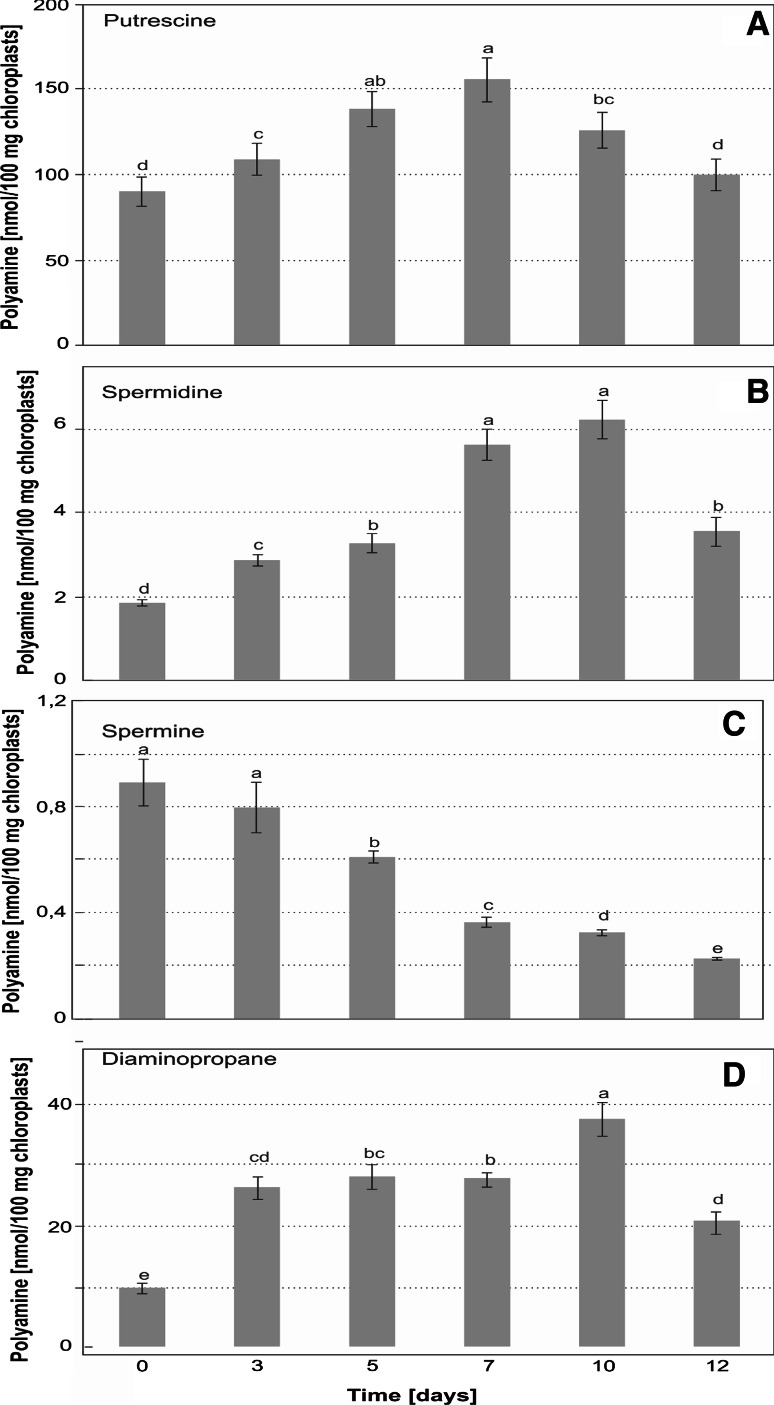
Changes in the level of plastid-associated polyamines: putrescine **(a)**, spermidine **(b)**, spermine **(c)** and diaminopropane **(d)** determined during dark-induced senescence of barley leaves. The differences in the measured parameters were analyzed for statistical significance using one-way analysis of variance (ANOVA) and the Tukey–Kramer Multiple Comparison Test (*n* = 9, *p* < *0.01*). The *same letter on bars* indicates that there was not significant differences between the means

The presence of ChlTGases in barley chloroplasts associated with the senescence progress indicates that the thylakoid membrane disassembly may be related to structural changes of plastid proteins mediated by polyamines. We therefore determined the levels of plastid membrane-associated polyamines: PU SD, SM and diaminopropane (DP, which is a product of oxidation of SD, SM, and indirectly also of PU), in each stage of leaf senescence (Fig. 2). The most abundant polyamine was PU and its level steadily increased until day 7 of senescence, when it reached a maximum level (170 % of the control level, measured at the senescence induction onset—day 0). Afterwards, we observed a gradual decrease in the level of PU, however, it was still above the control level by 38 % at day 10 and by 11 % at day 12 (Fig. 2a).

The control level of SD was about 47 times lower than PU and it increased dynamically during the chloroplast senescence, reaching its maximum level at day 10 (350 % of the control value). The highest increase was noticeable between days 0 and 3 as well as between days 5 and 7, with a rise of about 50 % between consecutive time points in each case. At day 12 the amount of SD dropped by about 42 % of its maximum level and was still much higher than in the control (Fig. 2b).

The level of SM was lower than PU by two orders of magnitude. Unlike other polyamines, the SM amount dropped throughout the entire period of leaves senescence, achieving 3.5 times reduction of its control level at the last analyzed time point. We observed the strongest reduction in the level of SM between days 5 and 7 (from 70 % down to 43 % of the control level) (Fig. 2c).

The amount of DP showed a step wise increase in the chloroplasts of senescing barley leaves, observable between days 0 and 3, when it reached 254 % of the control activity as well as between days 7 and 10, when it reached its maximum at 381 % of the control activity. Afterwards, the level of DP dropped but was still as much as 208 % of the control activity, at day 12 (Fig. 2d).

### Changes in the ChlTGase activity during senescence

**Fig. 3:**
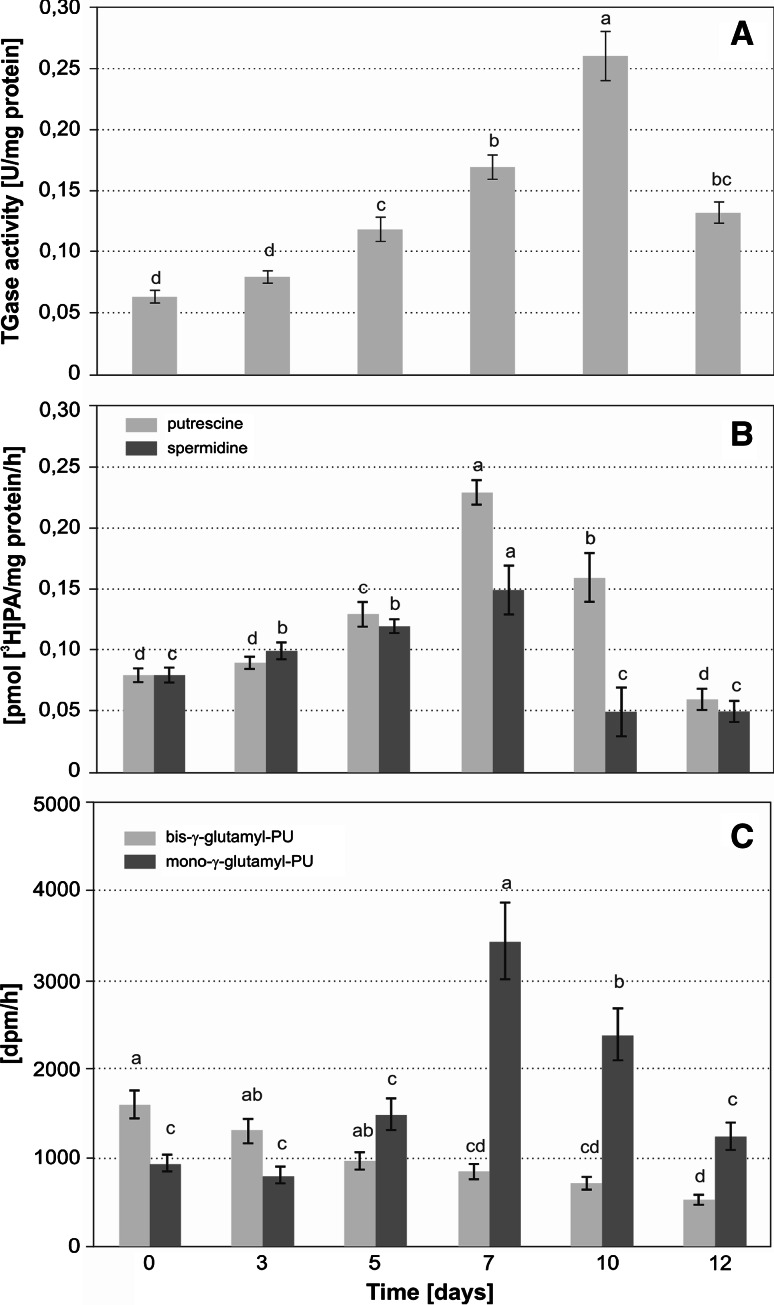
**a** biotin-cadaverine binding to *N,N′*-dimethylcasein (colorimetric assay), **b** [^3^H]PU and [^3^H]SD conjugation to plastid proteins (radiometric assay), **c** levels of mono- and bis-(γ-glutamyl)-[^3^H]PU derivatives. The differences in the measured parameters were analyzed for statistical significance using one-way analysis of variance (ANOVA) and Tukey–Kramer Multiple Comparison Test (**a**, *n* = 9, **b**, **c**, *n* = 6, *p* < *0.01*). The *same letter on bars* indicates that there was no significant differences between the means

Having confirmed the presence of ChlTGases and modulation of polyamine levels in senescing barley chloroplast, we turned to the analysis of barley ChlTGases activity. We used colorimetric and radiometric methods to monitor the changes in the ChlTGases total activity during dark-induced leaf senescence. In the first assay, the TGase enzymatic activity was measured as the ability to conjugate the exogenous substrates biotin-cadaverine to *N,N′*-dimethylcasein in a calcium-dependent manner. Using this assay, we observed a continuous increase in the TGase activity until day 10 of senescence, when it reached 0.26 U/mg protein (412 % of the activity measured on day 0) (Fig. 3a). After day 10, a rapid decline in the activity of the enzyme was observed, and the activity achieved 50 % of the maximal value on day 12.

In the second (radiometric) assay, the ChlTGase activity was estimated based on the rate of the radiolabeled polyamine conjugation to endogenous proteins. Our results suggested that the levels of PU and SD (but not SM) bound to the thylakoid fractions of the barley leaves increased during the early stages of barley leaf senescence. We therefore conducted a time course of the changes in the levels of [^3^H]PU or [^3^H]SD incorporation into the endogenous plastid proteins to ascertain which of the two polyamines is most efficiently conjugated by the ChlTGases during senescence (Fig. 3b). The amounts of bound [^3^H]PU and [^3^H]SD were equal before the induction of the senescence process and did not significantly change after 3 days of incubation in the darkness. A significant increase in the polyamine conjugation was observed on day 5 and reached a maximum on day 7; however, the [^3^H]PU peak was much higher—more than 270 % of the control level (day 0)—while the maximal amount of bound [^3^H]SD was approximately 200 % of the control. On day 10, the level of the conjugated SD dropped below the level of the control, while the level of the conjugated PU remained approximately two times higher than it was before the onset of senescence and eventually decreased below the control level on day 12.

PU was revealed to be the major polyamine involved in the post-translational modifications of the plastid proteins, and we therefore analyzed the distribution of protein-bound mono- and bis-(γ-glutamyl)-PU derivatives in the plastids of the senescing barley leaves (Fig. 3c). The senescence-associated changes in the amount of mono-(γ-glutamyl)-PU mirrored those observed for total PU conjugation (presented in Fig. 3b), with a strong peak on day 7 (365 % of the control level), and a subsequent decrease on days 10–12. At the same time, the level of bis-(γ-glutamyl)-PU, which was the dominant PU derivative on day 0, continually decreased through the entire senescence period to approximately 45 % of the initial amount.

### Plastid proteins as TGase substrates

**Fig. 4:**
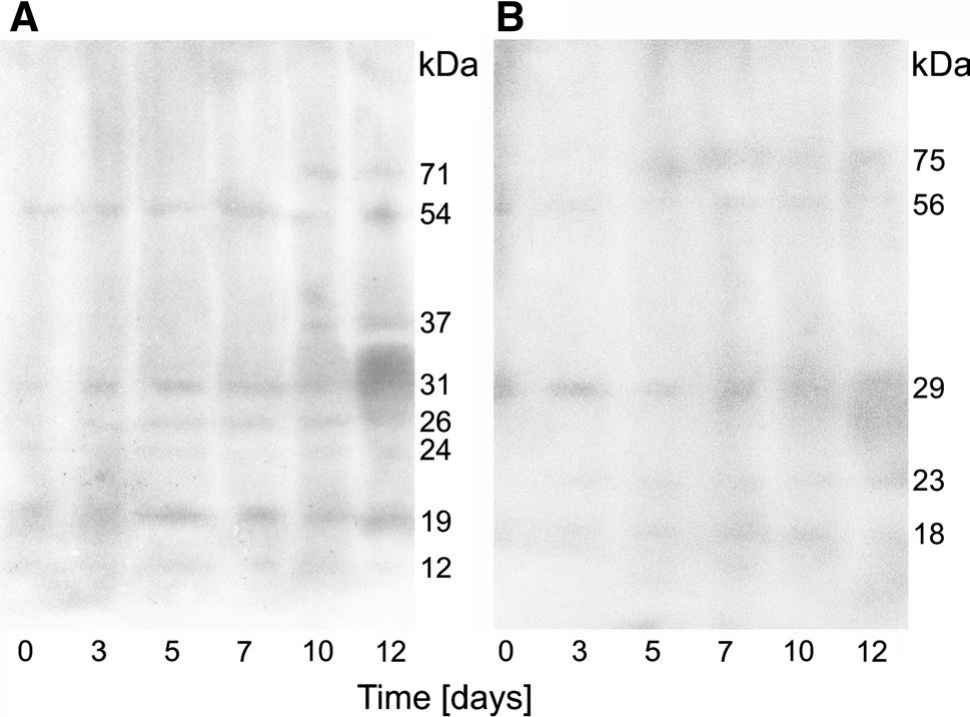
Autoradiography of SDS gel electrophoresis of barley plastid proteins isolated from dark-induced senescing leaves and incubated in the presence of [^3^H]PU (**a**) and [^3^H]SD (**b**)

Once the TGase catalytic activity was detected in senescing chloroplasts and we confirmed the presence of physiological protein substrates for ChlTGases, we aimed to identify the plastid proteins modified by ChlTGase-mediated cross-linking. The autoradiography of the SDS-PAGE separated plastid proteins, which were bound to [^3^H]PU and [^3^H]SD, revealed 8 and 5 protein bands, respectively (71, 54, 37, 31, 26, 24, 19, and 12 kDa for PU and 75, 56, 29, 23, and 18 kDa for SD; see Fig. 4). Both polyamines were conjugated to multiple proteins that are involved in the organization and regulation of the photosystem apparatus (details of the MS/MS identification of the protein bands are presented in Table 1) and were detected in all time points, including in the control. Histone-like protein (12 kDa) was observed from days 0–7, whereas peroxiredoxin and class I heat shock protein (23 and 18 kDa, respectively) were not detected before senescence induction and appeared as TGase substrates, beginning on day 3. IAA (indoleacetic acid)-amino acid hydrolase (75 kDa) was observed beginning on day 5 and two proteins, starch synthase (71 kDa) and ent-copalyl diphosphate synthase (37 kDa), were visible only in the late senescence stages (beginning on day 10).

**Table 1:** MS/MS identification of [^3^H]PA-conjugated proteins in senescing barley leaves

Band mass (kDa)^a^	Best protein homologue				
	NCBI/Swiss-Prot ID	Description	Function^b^	Mass (kDa)	Score
71	Q43654.2/SSY1_WHEAT	Starch synthase 1, chloroplastic/amyloplastic, *Triticum aestivum*	Glycan biosynthesis; starch biosynthesis. Belongs to the glycosyltransferase 1 family. Bacterial/plant glycogen synthase subfamily	71.5	65
54	P00828.2/ATPB_HORVU	ATP synthase subunit beta, chloroplastic, *Hordeum vulgare*	Produces ATP from ADP in the presence of a proton gradient across the membrane. The catalytic sites are hosted primarily by the beta subunits. Belongs to the ATPase alpha/beta chains family	53.9	176
37	O04408.1/KSA_PEA	Ent-copalyl diphosphate synthase, chloroplastic, *Pisum sativum*	Catalyzes the conversion of geranylgeranyl diphosphate to the gibberellin precursor ent-copalyl diphosphate. Belongs to the terpene synthase family	93.7	70
31	P0CJ48.1/CB1A_ARATH	Chlorophyll *a*–*b* binding protein 2, chloroplastic, *Arabodopsis thaliana*	The light-harvesting complex (LHC) functions as a light receptor, it captures and delivers excitation energy to photosystems with which it is closely associated. Belongs to the LHC protein family	28.3	48
26	P54773.1/PSBS_SOLLC	Photosystem II 22 kDa protein, chloroplastic, *Solanum lycopersicum*	Is involved in non-photochemical quenching, a process maintains the balance between dissipation and utilization of light energy to minimize generation of oxidizing molecules, thereby protecting the plant against photo-oxidative damage. Belongs to the ELIP/psbS family	29.2	81
24	P08963.1/CB22_HORVU	Chlorophyll *a*–*b* binding protein 2, chloroplastic, *Hordeum vulgare*	The light-harvesting complex (LHC) functions as a light receptor, it captures and delivers excitation energy to photosystems with which it is closely associated. Belongs to the LHC protein family	24.1	49
19	GI:131225/PSAL_HORVU	Photosystem I reaction center subunit XI, chloroplastic, *Hordeum vulgare*	A photochemical system containing P700, the chlorophyll a dimer that functions as a primary electron donor. Functioning as a light-dependent plastocyanin-ferredoxin oxidoreductase, it transfers electrons from plastocyanin to ferredoxin. Belongs to the PsaL family	22.3	75
12	P02275.2/H2A1_WHEAT	Histone H2A.1, *Triticum aestivum*	Histone-like protein of chloroplast. Limiting DNA accessibility to the cellular machineries which require DNA as a template. Histones thereby play a central role in transcription regulation, DNA repair, DNA replication and stability	15.6	98
75	Q8H3C7.2/ILL9_ORYSJ	IAA-amino acid hydrolase ILR1-like 9, *Oryza sativa*	Hydrolyzes certain amino acid conjugates of the plant growth regulator indole-3-acetic acid. Belongs to the peptidase M20 family	46.3	48
56	P10900.2/PSBB_HORVU	Photosystem II CP47 chlorophyll apoprotein, *Hordeum vulgare*	One of the components of the core antenna complex of photosystem II. It binds chlorophyll and helps catalyze the primary light-induced photochemical processes of photosystem II. Belongs to the PsbB/PsbC family	56.1	228
29	Q00434.1/PSBP_WHEAT	Oxygen-evolving enhancer protein 2, chloroplastic, *Triticum aestivum*	Is involved in the regulation of photosystem II. Associated with the photosystem II complex. Belongs to the psbP family	27.2	65
23	Q96468.1/BAS1_HORVU	2-Cys peroxiredoxin BAS1 chloroplastic, *Hordeum vulgare*	An antioxidant enzyme. Belongs to the AhpC/TSA family	23.4	74
18	Q9XIE3.1/HS17A_ARATH	17.6 kDa class I heat shock 1, *Arabidopsis thaliana*	Possesses chaperone activity. Belongs to the small heat shock protein (HSP20) family	17.6	45

Best protein homologue was identified with Mascot in NCBI/SwissProt databases

^a^Molecular mass of each protein band visualised by autoradiography, as reported in Fig. 4

^b^Functional annotation according to REVIGO, GenomeNet and UniProt

### Identification and expression analysis of *AtPng1p* homologue in barley

To date, the cDNA sequences of two apparently non-orthologous genes whose products possess TGase activity (as documented by in vitro or in vivo experiments) have been described in plants. The first gene codes for the *Arabidopsis* AtPng1p protein (TAIR locus AT5G49570) (Della Mea et al. 2004a). The other report concerns the highly similar *Zea mays* TGases TGZ15 and TGZ21, which were discovered by the immunoscreening of a cDNA library (Villalobos et al. 2004). Considering that over 22,500 full-length cDNAs that were derived from various barley cDNA libraries have been recently added to available sequence resources (Matsumoto et al. 2011), a computational search for barley proteins with a high homology to AtPng1p or TGZ15/TGZ21 proteins was performed (Supplementary Text, Supplementary Figures S1–S6, Supplementary Table S1). As a result, a 777 aa long protein exhibiting a high degree of similarity to the AtPng1p reference protein sequence has been identified. It possesses a conserved core domain with a catalytic triad, characteristic of TGases. Throughout the article, we will refer to this protein as HvPng1-like. As we were not able to identify any protein homologue of maize TGZ15 and TGZ21 in the public databases nor did we find the corresponding coding sequence in the reference maize genomic DNA sequence, we focused on the functional analysis of only the *HvPng1*-*like* gene.

**Fig. 5:**
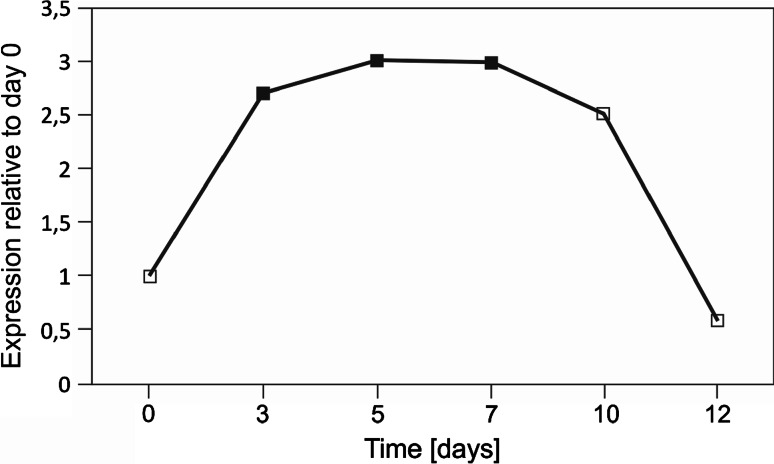
The time course of *HvPng1*-*like* expression during the 12 days of senescence (data averaged from four biological replicates). Significance of the relative changes in gene expression (compared to day 0) was tested with Pair Wise Fixed Reallocation Randomisation Test (Pfaffl et al. 2002). Significant changes were observed at day 3 (*p* = 0.001), day 5 (*p* = 0.003), and day 7 (*p* = 0.002) and are marked as *black* *filled* *squares*

In barley, *HvPng1*-*like* is expressed at a medium level and does not show major fluctuations throughout the plant development (Druka et al. 2006) (Supplementary Figure S7). We analyzed expression changes of the *HvPng1*-*like* gene during the dark-induced senescence of barley leaves in four independent biological replicates. We repeatedly observed increase in *HvPng1*-*like* transcript amount, associated with the senescence progress, although the level and the duration time of gene induction varied between the replicates (Supplementary Figure S8). The averaged profile of *HvPng1*-*like* expression is presented in Fig. 5. The level of transcript increased at the onset of the senescing process (day 3), remained elevated up to day 10, and then dropped on day 12, with significant changes observed on day 3, 5, and 7.

## Discussion

The senescence is a natural developmental phase in the life of plant. It can be also induced by various types of stress, leading to death of plant parts, for example leaf. The senescence proceeds through a series of synchronized events and is tightly regulated by a variety of internal factors, including plant hormones (Sarwat et al. 2013). Here we show that physiological and structural changes of chloroplasts, associated with the dark-induced senescence of barley leaves, involve polyamine conjugation and modification of chloroplast proteins, accompanied by modulation of barley ChlTGases localization and activity. Earlier studies of senescing barley leaves showed that the level of the 78 kDa TGase was lower when the samples were incubated with cytokinin, a phytohormone known for its anti-senescence properties (Sobieszczuk-Nowicka et al. 2009). Using an anti-TGase antibody, we detected three polypeptides in the chloroplasts of senescing barley leaves with the following masses 33, 58, and 78 kDa (Sobieszczuk-Nowicka et al. 2009). This conclusion is now supported by suborganellar detection of ChlTGases. The plastid enzyme might be involved in the stabilization of the plastid structure under stress conditions (darkness) and/or in its rearrangement during the chloroplast-to-gerontoplast transformation, similar to how it contributes to the conversion of an etioplast into a chloroplast (Sobieszczuk-Nowicka et al. 2008).

An increase in the activity of TGase was observed until day 7, in the presence of [^3^H]PU and [^3^H]SD. The upward trend (until day 10) was also observed during the colorimetric analysis of the ChlTGase activity. The shift of the maximum enzyme activity peak between the two methods most likely resulted from the different availability of protein substrates for the enzyme. When measuring the TGase activity using the radiometric method, the enzyme recognized the endogenous plastid proteins and not the exogenously applied substrate, as in the colorimetric assay. Therefore, as the degradation of the chloroplast structures advanced, a decrease in the plastid proteins might have been the limiting factor for the later time points (days 10 and 12) of the radiometric assay. Moreover, the senescence-associated changes in the amount of mono- and bis-(γ-glutamyl)-PU corroborated the studies of the Serafini-Fracassini team run on tobacco corolla which showed that during petals senescence the decrease in the bis-(γ-glutamyl)-PU and bis-(γ-glutamyl)-SD occurs, whereas the amount of mono-(γ-glutamyl)-PU increased (Serafini-Fracassini et al. 2002).

In the senescing barley leaves, changes in ChlTGase amount and localization paralleled the changes in the level of plastid membrane-bound polyamines: PU and SD, the levels of which continuously increased till day 7–10 of the process. Polyamines, which are low molecular weight cations synthesized in almost all biological systems, are implicated in a divergent array of processes and are believed to be important modulators of plant growth and development (Walden et al. 1997). Their intracellular titers must be strictly regulated (Moschou et al. 2008; Takahashi and Kakehi 2010). High polyamine levels have been observed in actively growing tissues (Mattoo et al. 2010), while their amount was suppressed in senescing tissues, both in the developmental and stress-induced senescence processes (Altman and Bachrach 1981; Sobieszczuk-Nowicka et al. 2009).

An increase in TGase activity, in cooperation with enhanced polyamine biosynthesis may result in the modification of a protein charge and conformation through the formation of cross-links within the same protein or between two or more proteins, which are then active in senescence. These cross-links contribute to the stability of protein structures and changes in their solubility, and ability to interact with other molecules (Lorand and Graham 2003; Serafini-Fracassini and Del Duca 2008; Serafini-Fracassini et al. 2009). Conversely, the modification or change in the protein availability elucidated by initiated organelle degradation could affect the PU and SD linkage, with a possible impact on the protein activity and senescence progress. Regardless of the mechanism involved, the detection of polyamines (PU and SD) covalently linked to glutamyl residues of specific endogenous proteins demonstrates the selectivity of TGase action; therefore, a physiological role might be ascribed to this enzyme in the senescence context. The MS/MS identification of the proteins bound to [^3^H]PU and [^3^H]SD showed that the protein components of both photosystems were modified by ChlTGase activity. The apoproteins of the chlorophyll *a*/*b* antenna complex have already been suggested as the substrates of ChlTGases in mature leaves (Del Duca et al. 1994; Della Mea et al. 2004b; Dondini et al. 2003). Recently, the LC–MS mass spectrometry identification of protein complexes indicated also that TGZ proteins form part of a specific photosystem II protein complex, which includes LHCII, ATPase, and pSbS, that plays a role in energy-dependent quenching that increases thermal dissipation of excess absorbed light energy in the photosystem (Campos et al. 2010).

The other identified protein, histone, which is also known animal TGase substrate (Ballestar et al. 1996), may correspond to the chloroplast histone-like protein (Kobayashi et al. 2002; Melonek et al. 2012). In accordance with the presence of histone-like proteins in plastids, in recent years, evidence about these proteins involves in regulation of plastid nucleoids morphology and compactness have been accumulated. Histone-like chloroplast protein can be either coded by the chloroplast DNA (Kobayashi et al. 2002) or by nuclear DNA and, which was found by fusion with GFP, to be dually targeted: to plastids and to the nucleus (Melonek et al. 2012). This result would suggest a TGase contribution in a well-known role of polyamines in DNA repair, replication or stability (Sobieszczuk-Nowicka and Legocka 2014). Most of all, we identified several stress-response proteins in the polyamine-bound fraction. The antioxidant enzymes peroxiredoxin, heat shock protein, ent-copalyl diphosphate synthase, are involved in gibberellin biosynthesis and IAA-amino acid hydrolase (Cejudo et al. 2012; Noushina et al. 2011; Van der Graaff et al. 2006; Wang et al. 2004).

None of those proteins were detected in the polyamine-bound fraction before the induction of the senescence process. A combination of the proteomic data and the results of the TGase activity assays support our hypothesis that the ChlTGases and polyamines are functionally involved in the cellular mechanisms of senescence. These data also provide a logical explanation of our previous observations: the addition of the anti-senescing hormone kinetin inhibited the dark-induced increase of ChlTGase activity and inhibited the increase of polyamine accumulation in the barley thylakoid membrane fraction (Sobieszczuk-Nowicka et al. 2009).

The only membrane-bound polyamine whose level decreased during senescence was SM. Its decreasing level during chloroplast degradation could be related to the breakdown of chloroplasts and degradation of proteins of the chlorophyll *a*/*b* antenna complexes to which it is bound. The released SM can be converted to polyamines with a lower molecular mass, such as PU and SD (Serafini-Fracassini et al. 2010). It also cannot be ruled out that as a result of oxidative deamination SM, but also other polyamines, are the source of H_2_O_2_ and/or cytotoxic aldehydes, e.g. acroleins (Novakoudis et al. 2007; Takahashi and Kakehi 2010). Polyamine analysis showed an increasing level of DP. Its presence, therefore, indicates the occurrence of oxidation processes that might contribute to chloroplast degradation. It has been also suggested that mono-derivative polyamines are preferred substrates for oxidases (Serafini-Fracassini et al. 2002). In this case, TGases could also indirectly stimulate the formation of reactive oxygen species in the chloroplasts and affect or execute their degradation.

The activity of ChlTGases through senescence can be controlled not only by the post-translational regulation, but also by transcription. The most studied plant gene coding for a protein with TGase activity is Arabidopsis *AtPng1p*. It has been shown that in conditions of undisturbed growth, the *AtPng1p* gene undergoes constitutive expression at a low level in all plant organs during various stages of development and under various light conditions (Della Mea et al. 2004a). We observed the same expression pattern for the *HvPng1*-*like* gene, which was computationally identified on the basis of its high sequence homology to *AtPng1p*. We also analyzed its expression profile during plant senescence. Our real-time RT-PCR experiments showed that expression of *HvPng1*-*like* increased as early as day 3 of senescence, when the mechanisms associated with the degradation of cell structures begin to be activated, and was up-regulated until day 10.

In our analysis, we were not able to identify any plant cDNA/protein sequence in the public databases with full-length amino acid sequence homology to TGZ15/TGZ21, two other TGases described previously (Villalobos et al. 2004). Despite high homology on the nucleotide level, the unique deletion of G in the *TGZ15/TGZ21* cDNA sequences resulted in a frameshift in their amino acid sequences, and consequently, a lack of homology at their C-termini, where the TGase catalytic triad was found by Villalobos et al. (2004). Recently, the same group reported the isolation and characterization of a rice homolog of the TGZ15/TGZ21 proteins named TGO. The TGO cDNA possesses a unique G deletion not observed in the other available rice sequences, including the genomic sequence of rice chromosome 4 (Campos et al. 2013). The maize TGZ15/TGZ21 and rice TGO proteins share high sequence homology (70 %), but only upstream of the G deletion positions, while their C-termini differ in length and sequence as well as in catalytic triad localization. However, overexpression experiments provide evidence that both proteins possess TGase activity (Campos et al. 2010, 2012, 2013; Ioannidis et al. 2009, 2012; Ortigosa et al. 2010; Villalobos et al. 2004), although their catalytic domains are not conserved.

## Conclusions 

In this study, we presented experimental evidence that the barley ChlTGase is activated during the dark-induced leaf senescence, which corresponds to the enhanced local TGase accumulation and activity and the increased expression level of the barley *HvPng1*-*like* gene. The ChlTGase localization within chloroplast structures, as well as the identification of post-translational modification of plastid proteins (polyamine-conjugated proteins), suggests a wide contribution of ChlTGases to dark-induced senescence-associated responses, including the stress response, inhibition of photosynthesis, and cell death manifested by the chloroplast-to-gerontoplast conversion and degradation. Together, the presented results deepen our knowledge of the mechanisms of the events happening in dark-induced senescence of barley leaves.

## Electronic supplementary material

Below is the link to the electronic supplementary material.

Supplementary material 1 (PDF 444 kb)

## Abbreviations

*DP*: Diaminopropane
*ChlTGase*: Chloroplast transglutaminase
*CPs*: Proteins of chlorophyll-protein complexes transducing excitation energy
*LHCII*: Antenna proteins of light-harvesting chlorophyll *a*/*b*-protein complex
*PU*: Putrescine
*REST*: Relative expression software tools
*SD*: Spermidine
*SM*: Spermine
*TGase*: Transglutaminase
