# Supplementary material for: Dark-induced senescence of barley leaves involves activation of plastid transglutaminases
Source: Amino Acids. 2015 Jan 13;47(4):825–38. doi: 10.1007/s00726-014-1912-y (PMC4361728; doi:10.1007/s00726-014-1912-y)

Supplementary Text

Computational identification of putative barley TGases

Coding sequences of putative barley TGase genes were searched for using Arabidopsis AtPng1p (Della Mea et al. 2004a)) and maize TGZ15 and TGZ21 (Villalobos et al. 2004) protein sequences to query the public databases. One predicted barley protein has been identified (BAK01337.1, hereafter named HvPng1-like) as exhibiting a high degree of similarity to the AtPng1p reference protein sequence. The corresponding cDNA sequence was successfully verified by assembling it with eight ESTs from the NCBI UniGene Hv.16669 (Figure S1). HvPng1-like has a high level of similarity to multiple proteins from both monocots and dicots (Table S1), all of which possess a conserved core domain (pfam01841) and whose Cys-His-Asp residues form the catalytic triad, a characteristic of TGases (Figure S2). According to the TargetP1.1 Server search, none of these proteins possess a classical signal peptide capable of targeting it to any cellular compartment.

When the maize TGZ21 protein was used for the identification of barley homologs, four predicted proteins with partial homology were identified, BAJ92539.1 being the most similar. We refer to this putative protein as HvTGZ21-like, whose cDNA sequence has been confirmed by assembly with 18 other cDNAs/ESTs from the NCBI UniGene Hv.1565 (Figure S3). Interestingly, HvTGZ21-like shows a very high degree of similarity to TGZ21 at the N-terminus, but the C-termini of the two proteins are drastically different. A more detailed investigation revealed that although numerous monocot cDNA sequences are highly similar to the *TGZ15* and *TGZ21* cDNA sequences, these two maize cDNAs lack a conserved G in position 747, which results in a frame shift that extends the TGZ15/TGZ21 protein length, with respect to the other *Poaceae* sequences (Figures S4 and S5). According to Villalobos et al. (2004), the TGase catalytic triad in the TGZ15 and TGZ21 proteins is placed in this unique C-terminal region. As we did not find any proteins with similarity to this region in the databases nor did we observe the G deletion in any other maize DNA sequence (including genomic DNA; see Figure S6), we focused on the functional analysis of only the *HvPng1-like* gene.

Table S1. Plant proteins highly similar to barley HvPng1-like protein.

Organism	NCBI accession	Length	Identities	Positives
H. vulgare **(HvPng1-like)**	**BAK01337**	**777 aa**	**-**	**-**
O. sativa	EEC82091	802 aa	76 %	83 %
B. distachyon	XP_003563069	719 aa	75 %	81 %
S. bicolor	XP_002462888	727 aa	70 %	79 %
R. communis	XP_002512221	719 aa	52 %	70 %
S. lycopersicum	NP_001234560	725 aa	53 %	69 %
V. vinifera	XP_002278422	726 aa	51 %	66 %
M. truncatula	XP_003626428	719 aa	49 %	66 %
A. thaliana (AtPng1p)	NP_199768	721 aa	51 %	66 %

Figure S1. CAP3 assembly of sequences from the UniGene Hv.16669. Sequence AK370136 is a cDNA corresponding to HvPNG1-like protein, acc. no BAK01337.1. Coding sequence has been highlighted.

```
******************* Contig 1 ********************
12/gb=AK370136+
                    9/gb=FD519350- is in 12/gb=AK370136+
                    7/gb=BJ479950+ is in 12/gb=AK370136+
                    6/gb=BU970594+ is in 12/gb=AK370136+
                    3/gb=AV938210+ is in 12/gb=AK370136+
                    8/gb=BJ485214- is in 12/gb=AK370136+
                    2/gb=AV937315+ is in 12/gb=AK370136+
                    1/gb=AV835569- is in 12/gb=AK370136+
11/gb=GH218900-

DETAILED DISPLAY OF CONTIGS
******************* Contig 1 ********************
                          .    :    .    :    .    :    .    :    .    :    .    :
12/gb=AK370136+       GGCGACGTGGTCCACAGAGATAACGCTATGGTGGCTCGCAGGTTCGTAGTCCGGCAGGCC
7/gb=BJ479950+                           ATAACGCTATGGTGGCTCGCAGGTTCGTAGTCCGGCAGGCC
3/gb=AV938210+                                 CTATGGTGGCTCGCAGGTTCGTAGTCCGGCAGGCC
                      ____________________________________________________________
consensus             GGCGACGTGGTCCACAGAGATAACGCTATGGTGGCTCGCAGGTTCGTAGTCCGGCAGGCC

                          .    :    .    :    .    :    .    :    .    :    .    :
12/gb=AK370136+       TCCGCCGGCGAGGAGCACGAGGTCGAGTACGACACGGACGACGGACTCGACGTCCTGCGC
7/gb=BJ479950+        TCCGCCGGNGAGGAGCACGAGGTCGAGTACGACACGGACGACGGACTCGACGTCCTGCGC
3/gb=AV938210+        TCCGCCGGCGAGGAGCACGAGGTCGAGTACGACACGGACGACGGACTCGACGTCCTGCGC
                      ____________________________________________________________
consensus             TCCGCCGGCGAGGAGCACGAGGTCGAGTACGACACGGACGACGGACTCGACGTCCTGCGC

                          .    :    .    :    .    :    .    :    .    :    .    :
12/gb=AK370136+       TTTCAGATCTTCTCGCTCACCTCCGTCCCACCCGACCTCCAGAAGATCGTAGTGGAAGCG
7/gb=BJ479950+        TTTCAGATCTTCTCGCTCACCTCCGTCCCACCCGACCTCCAGAAGATCGTAGTGGAAGCG
3/gb=AV938210+        TTTCAGATCTTCTCGCTCACCTCCGTCCCACCCGACCTCCAGAAGATCGTAGTGGAAGCG
                      ____________________________________________________________
consensus             TTTCAGATCTTCTCGCTCACCTCCGTCCCACCCGACCTCCAGAAGATCGTAGTGGAAGCG

                          .    :    .    :    .    :    .    :    .    :    .    :
12/gb=AK370136+       GATGGCTCCGTGGTCGACGACGGCACAGGCCTCGAGTCAATTCCCGATGGCCTGTGCCTC
7/gb=BJ479950+        GATGGCTCCGTGGTCGACGACGGCACAGGCCTCGAGTCAATTCCCGATGGCCTGTGCCTC
3/gb=AV938210+        GATGGCTCCGTGGTCGACGACGGCACAGGCCTCGAGTCAATTCCCGATGGCCTGTGCCTC
                      ____________________________________________________________
consensus             GATGGCTCCGTGGTCGACGACGGCACAGGCCTCGAGTCAATTCCCGATGGCCTGTGCCTC

                          .    :    .    :    .    :    .    :    .    :    .    :
12/gb=AK370136+       GTGTCTATTGACGAGGGGGAGGACGCGGACGCGGCTTCGGCTTCGGCGACGGCGCAGGAG
7/gb=BJ479950+        GTGTCTATTGACGAGGGGGAGGACGCGGACGCGGCTTCGGCTTCGGCGACGGCGCAGGAG
3/gb=AV938210+        GTGTCTATTGACGAGGGGGAGGACGCGGACGCGGCTTCGGCTTCGGCGACGGCGCAGGAG
                      ____________________________________________________________
consensus             GTGTCTATTGACGAGGGGGAGGACGCGGACGCGGCTTCGGCTTCGGCGACGGCGCAGGAG

                          .    :    .    :    .    :    .    :    .    :    .    :
12/gb=AK370136+       AAGTCCGACGAGGAACTGAAGAGGTTGATTGAGATCGAGGTGGAGGCGGATGGCTCCGTG
7/gb=BJ479950+        AAGTCCGACGAGGAACTGAAGAGGTTGATTGAGATCGAGGTGGAGGCGGATGGCTCCGTG
3/gb=AV938210+        AAGTCCGACGAGGAACTGAAGAGGTTGATTGAGATCGAGGTGGAGGCGGATGGCTCCGTG
2/gb=AV937315+                                                           GGCTCCGTG
                      ____________________________________________________________
consensus             AAGTCCGACGAGGAACTGAAGAGGTTGATTGAGATCGAGGTGGAGGCGGATGGCTCCGTG

                          .    :    .    :    .    :    .    :    .    :    .    :
12/gb=AK370136+       GTCGACGACGTCACCGACCTCAAGTCTATTTCCGAAGTCACCCGCCTCGTACCCATCGGC
7/gb=BJ479950+        GTCGACGACGTCACCGACCTCAAGTCTATTTCCGAAGTCACCCGCCTCGTACCCATCGGC
3/gb=AV938210+        GTCGACGACGTCACCGACCTCAAGTCTATTTCCGAAGTCACCCGCCTCGTACCCATCGGC
2/gb=AV937315+        GTCGACGACGTCACCGACCTCAAGTCTATTTCCGAAGTCACCCGCCTCGTACCCATCGGC
                      ____________________________________________________________
consensus             GTCGACGACGTCACCGACCTCAAGTCTATTTCCGAAGTCACCCGCCTCGTACCCATCGGC

                          .    :    .    :    .    :    .    :    .    :    .    :
12/gb=AK370136+       GAGGGGGATGACGTGGACGCAGCGGCGGCTGCAGCTATGAGGGCGCAGGAGAAGTCCGAC
7/gb=BJ479950+        GAGGGGGATGACGTGGACGCAGCGGCGGCTGCAGCTATGAGGGCGCAGGAGAAGTCCGAC
6/gb=BU970594+                                                 GGCGCAGGAGAAGTCCGAC
3/gb=AV938210+        GAGGGGGATGACGTGGACGCAGCGGCGGCTGCAGCTATGAGGGCGCAGGAGAAGTCCGAC
2/gb=AV937315+        GAGGGGGATGACGTGGACGCAGCGGCGGCTGCAGCTATGAGGGCGCAGGAGAAGTCCGAC
                      ____________________________________________________________
consensus             GAGGGGGATGACGTGGACGCAGCGGCGGCTGCAGCTATGAGGGCGCAGGAGAAGTCCGAC
```

```
                         .    :    .    :    .    :    .    :    .    :    .    :
12/gb=AK370136+     GAGGAGCTCACGAGGATGATTCAGGCGGAAGAAGAGGCACTTCTACTGCAAAAGTACAGC
7/gb=BJ479950+      GAGGAGCTCACGAGGATGATT
6/gb=BU970594+      GAGGAGCTCACGAGGATGATTCAGGCGGAAGAAGAGGCACTTCTACTGCAAAAGTACAGC
3/gb=AV938210+      GAGGAGCTCACGAGGATGATTCANGCGGAAGAAGAGGCACTTNTACTGCAAAAGTACAGC
2/gb=AV937315+      GAGGAGCTCACGAGGATGATTCAGGCGGAAGAAGAGGCACTTCTACTGCAAAAGTACAGC
                    ____________________________________________________________
consensus           GAGGAGCTCACGAGGATGATTCAGGCGGAAGAAGAGGCACTTCTACTGCAAAAGTACAGC

                         .    :    .    :    .    :    .    :    .    :    .    :
12/gb=AK370136+     ATACAAA-GTGACAGAGGAGAGGTGTTCAGAGAAAGAGTTGAGCCATACATGCGCCAGGT
6/gb=BU970594+      ATACAAA-GTGACAGAGGAGAGGTGTTCAGAGAAAGAGTTGAGCCATACATGCGCCAGGT
3/gb=AV938210+      ATACAAAAGTGACAGAGGAGAGGTGTTCANAGAAAGAGNTGAGCCATACATGCG
2/gb=AV937315+      ATACAAA-GTGACAGAGGAGAGGTGTTCAGAGAAAGAGTTGAGCCATACATGCGCCAGGT
                    ____________________________________________________________
consensus           ATACAAA-GTGACAGAGGAGAGGTGTTCAGAGAAAGAGTTGAGCCATACATGCGCCAGGT

                         .    :    .    :    .    :    .    :    .    :    .    :
12/gb=AK370136+     TCTGAAGTACGAGGATTCGGTGCATCAGGAAGCAGCTCTGAAGACGGTTCCTGTAGATGA
6/gb=BU970594+      TCTGAAGTACGAGGATTCGGTGCATCAGGAAGCAGCTCTGAAGACGGTTCCTGTAGATGA
2/gb=AV937315+      TCTGAAGTACGAGGATTCGGTGCATCAGGAAGCAGCTCTGAAGACGGTTCCTGTAGATGA
                    ____________________________________________________________
consensus           TCTGAAGTACGAGGATTCGGTGCATCAGGAAGCAGCTCTGAAGACGGTTCCTGTAGATGA

                         .    :    .    :    .    :    .    :    .    :    .    :
12/gb=AK370136+     ACTTAAGGAGAAGGCGTTGATTTCACTGGCCAAGGAAGGGACCTTCAGTCCTTCGAAAAA
6/gb=BU970594+      ACTTAAGGAGAAGGCGTTGATTTCACTGGCCAAGGAAGGGACCTTCAGTCCTTCGAAAAA
2/gb=AV937315+      ACTTAAGGAGAAGGCGTTGATTTCACTGGCCAAGGAAGGGACCTTCAGTCCTTCGAAAAA
                    ____________________________________________________________
consensus           ACTTAAGGAGAAGGCGTTGATTTCACTGGCCAAGGAAGGGACCTTCAGTCCTTCGAAAAA

                         .    :    .    :    .    :    .    :    .    :    .    :
12/gb=AK370136+     TGAAGAGGATCATGCCTTTCTGTTGGAATTGCTTTTCTGGTTTAAGCAATCATTCAGATG
6/gb=BU970594+      TGAAGAGGATCATGCCTTTCTGTTGGAATTGCTTTTCTGGTTTAAGCAATCATTCAGATG
2/gb=AV937315+      TGAAGAGGATCATGCCTTTCTGTTGGAATTGCTTTTCTGGTTTAAGCAATCATTCAGATG
                    ____________________________________________________________
consensus           TGAAGAGGATCATGCCTTTCTGTTGGAATTGCTTTTCTGGTTTAAGCAATCATTCAGATG

                         .    :    .    :    .    :    .    :    .    :    .    :
12/gb=AK370136+     GGTTAATGCAGCAGCTTGTGACATCTGTGACCGTGAAACATCTATGATTGGAATGGGAAA
6/gb=BU970594+      GGTTAATGCAGCAGCTTGTGACATCTGTGACCGTGAAACATCTATGATTGGAATGGGAAA
2/gb=AV937315+      GGTTAATGCAGCAGCTTGTGACATCTGTGACCGTGAAACATCTATGATTGGAATGGGAAA
                    ____________________________________________________________
consensus           GGTTAATGCAGCAGCTTGTGACATCTGTGACCGTGAAACATCTATGATTGGAATGGGAAA

                         .    :    .    :    .    :    .    :    .    :    .    :
12/gb=AK370136+     CCCACTTCCCGCAGAAATTGAATTTGGTGCTTCGCGTGTCGAGATCTATAGGTGCAACCA
6/gb=BU970594+      CCCACTTCCCGCAGAAATTGAATTTGGTGCTTCGCGTGTCGAGATCTATAGGTGCAACCA
2/gb=AV937315+      CCCACTTCCCGCAGAAATTGAATTTGGTGCTTCGCGTGTCGAGATCTATAGGTGCAACCA
                    ____________________________________________________________
consensus           CCCACTTCCCGCAGAAATTGAATTTGGTGCTTCGCGTGTCGAGATCTATAGGTGCAACCA

                         .    :    .    :    .    :    .    :    .    :    .    :
12/gb=AK370136+     TTGTTCGAGCATCATCCGTTTCCCAAGGTACAATGATCCGCATAAGCTTATACAAACTAG
6/gb=BU970594+      TTGTTCGAGCATCATCCGTTTCCCAAGGTACAATGATCCGCATAAGCTTATACAAACTAG
2/gb=AV937315+      TTGTTCGAGCATCATCCGTTT
                    ____________________________________________________________
consensus           TTGTTCGAGCATCATCCGTTTCCCAAGGTACAATGATCCGCATAAGCTTATACAAACTAG

                         .    :    .    :    .    :    .    :    .    :    .    :
12/gb=AK370136+     GAAAGGACGCTGTGGAGAATGGGCAAACTGCTTCACATTCTACTGTCGAGTTTTTGGATA
6/gb=BU970594+      GAAAGGACGCTGTGGAGAATGGGCAAACTG
                    ____________________________________________________________
consensus           GAAAGGACGCTGTGGAGAATGGGCAAACTGCTTCACATTCTACTGTCGAGTTTTTGGATA

                         .    :    .    :    .    :    .    :    .    :    .    :
12/gb=AK370136+     CGAAGCTCGTTTGATTCTGGATTTCACTGATCATGTTTGGACAGAATGTTTTTCAAACCT
                    ____________________________________________________________
consensus           CGAAGCTCGTTTGATTCTGGATTTCACTGATCATGTTTGGACAGAATGTTTTTCAAACCT

                         .    :    .    :    .    :    .    :    .    :    .    :
12/gb=AK370136+     CTATGGGAGATGGATTCACTTCGATCCATGCGAAGGAGTCTATGATAATCCCTTGCTGTA
                    ____________________________________________________________
consensus           CTATGGGAGATGGATTCACTTCGATCCATGCGAAGGAGTCTATGATAATCCCTTGCTGTA

                         .    :    .    :    .    :    .    :    .    :    .    :
```

```
12/gb=AK370136+       TGAGAAAGGGTGGAACAAGAAATTAGATTATGCAATTGGTATCTCAAAAGATGGAGTACA

consensus             TGAGAAAGGGTGGAACAAGAAATTAGATTATGCAATTGGTATCTCAAAAGATGGAGTACA

                          .    :    .    :    .    :    .    :    .    :    .    :
12/gb=AK370136+       TGATGTAACTAAACGCTACACCAGAAAGTGGCATGAGGTTCTCTCTAGGCGGACCATTAC

consensus             TGATGTAACTAAACGCTACACCAGAAAGTGGCATGAGGTTCTCTCTAGGCGGACCATTAC

                          .    :    .    :    .    :    .    :    .    :    .    :
12/gb=AK370136+       CTCCGAGGATACAGTTTCAGCTATTCTAATGAATATAACTACAAAGTGTCGCAGTGGATT

consensus             CTCCGAGGATACAGTTTCAGCTATTCTAATGAATATAACTACAAAGTGTCGCAGTGGATT

                          .    :    .    :    .    :    .    :    .    :    .    :
12/gb=AK370136+       GTCAAGTGATGAACTCTTAGCCTTAGAAAACCGTGACAGGAAAGAGTCCAAGGAACTTAG

consensus             GTCAAGTGATGAACTCTTAGCCTTAGAAAACCGTGACAGGAAAGAGTCCAAGGAACTTAG

                          .    :    .    :    .    :    .    :    .    :    .    :
12/gb=AK370136+       TAAAGCTATCTATCTTGAAATCAACAACAGCATATCTCTACCTGGAAGGCAAAGTGGTTC

consensus             TAAAGCTATCTATCTTGAAATCAACAACAGCATATCTCTACCTGGAAGGCAAAGTGGTTC

                          .    :    .    :    .    :    .    :    .    :    .    :
12/gb=AK370136+       TGTGGAATGGAGAACAGCAAGGTCAGAATTGGGCCAAGCAGACTCACTCAGTTGCTCGTC

consensus             TGTGGAATGGAGAACAGCAAGGTCAGAATTGGGCCAAGCAGACTCACTCAGTTGCTCGTC

                          .    :    .    :    .    :    .    :    .    :    .    :
12/gb=AK370136+       GTGTCCTATTCGGAGATGTGTGGATGCTCATGTTTCAAAAATATATGATGCTCTTTCAGC

consensus             GTGTCCTATTCGGAGATGTGTGGATGCTCATGTTTCAAAAATATATGATGCTCTTTCAGC

                          .    :    .    :    .    :    .    :    .    :    .    :
12/gb=AK370136+       TATTCTTTCTTGTTTTTGTGATAATAACATCCGTAATGAAAGAATCATTGAAGTTTTCGT

consensus             TATTCTTTCTTGTTTTTGTGATAATAACATCCGTAATGAAAGAATCATTGAAGTTTTCGT

                          .    :    .    :    .    :    .    :    .    :    .    :
12/gb=AK370136+       CACATTGAGGAGCTTGATGCAAAACCTTAAAGATGCCAACTTCAAAAGCAGGAGAGTCAC

consensus             CACATTGAGGAGCTTGATGCAAAACCTTAAAGATGCCAACTTCAAAAGCAGGAGAGTCAC

                          .    :    .    :    .    :    .    :    .    :    .    :
12/gb=AK370136+       ATTAGATCAAAAATCGCAGCAAATTTTTGAGGAGATACTTCCCTCTGCTGAAAGATTGCT
1/gb=AV835569-                                                             GAAAGATTGCT

consensus             ATTAGATCAAAAATCGCAGCAAATTTTTGAGGAGATACTTCCCTCTGCTGAAAGATTGCT

                          .    :    .    :    .    :    .    :    .    :    .    :
12/gb=AK370136+       TTCTGCAGTTTCTTTGAAAGCAGACTTACACAATGATGGAGATCCATCCGTGGCTACAGA
1/gb=AV835569-        TTCTGCAGTTTCTTTGAAAGCAGACTTACACAATGATGGAGATCCATCCGTGGCTACAGA

consensus             TTCTGCAGTTTCTTTGAAAGCAGACTTACACAATGATGGAGATCCATCCGTGGCTACAGA

                          .    :    .    :    .    :    .    :    .    :    .    :
12/gb=AK370136+       TGGAAATCTAATACATACATCCTTAGCATTACCAGTAGCACTAGATGCAGTTGATGAGAT
1/gb=AV835569-        TGGAAATCTAATACATACATCCTTAGCATTACCAGTAGCACTAGATGCAGTTGATGAGAT

consensus             TGGAAATCTAATACATACATCCTTAGCATTACCAGTAGCACTAGATGCAGTTGATGAGAT

                          .    :    .    :    .    :    .    :    .    :    .    :
12/gb=AK370136+       ACTAAGTAACTATAAGAGCAATATCTTTTACACAAAAGGTCATCAATTTCCTAGAGGCAA
8/gb=BJ485214-                                         AAAAGGTCATCAATTTCCTAGAGGCAA
1/gb=AV835569-        ACTAAGTAACTATAAGAGCAATATCTTTTACACAAAAGGTCATCAATTTCCTAGAGGCAA

consensus             ACTAAGTAACTATAAGAGCAATATCTTTTACACAAAAGGTCATCAATTTCCTAGAGGCAA

                          .    :    .    :    .    :    .    :    .    :    .    :
12/gb=AK370136+       TAGACTCTGTTCAGGTTCAGTCCTTGCAAGCTGTGAGCAGCTCCCAATTGGAATTGCAAC
9/gb=FD519350-                                             CAGCTCCCAATTGGAATTGCAAC
8/gb=BJ485214-        TAGACTCTGTTCAGGTTCAGTCCTTGCAAGCTGTGAGCAGCTCCCAATTGGAATTGCAAC
1/gb=AV835569-        TAGACTCTGTTCAGGTTCAGTCCTTGCAAGCTGTGAGCAGCTCCCAATTGGAATTGCAAC

consensus             TAGACTCTGTTCAGGTTCAGTCCTTGCAAGCTGTGAGCAGCTCCCAATTGGAATTGCAAC
```

```
                            .    :    .    :    .    :    .    :    .    :    .    :
12/gb=AK370136+         AGCAGCCTTTGACGGCATTCGCTTGTCCAAATGGGAAGAACCTGATGGAGCCAAAGGATG
9/gb=FD519350-          AGCAGCCTTTGACGGCATTTGCTTGTCCAAATGGGAAGAACCTGATGGAGCCAAAGGATG
8/gb=BJ485214-          AGCAGCCTTTGACGGCATTCGCTTGTCCAAATGGGAAGAACCTGATGGAGCCAAAGGATG
1/gb=AV835569-          AGCAGCCTTTGACGGCATTCGCTTGTCCAAATGGGAAGAACCTGATGGAGCCAAAGGATG
                        ____________________________________________________________
consensus               AGCAGCCTTTGACGGCATTCGCTTGTCCAAATGGGAAGAACCTGATGGAGCCAAAGGATG

                            .    :    .    :    .    :    .    :    .    :    .    :
12/gb=AK370136+         TTGGCTAATGTACAAGGTGCGTGATGGCCAAACTTGTGAGTTGGAGTCATATGATTTGAT
9/gb=FD519350-          TTGGCTAATGTACAAGGTGCGTGATGGCCAAACTTGTGAGTTGGAGTCATATGATTTGAT
8/gb=BJ485214-          TTGGCTAATGTACAAGGTGCGTGATGGCCAAACTTGTGAGTTGGAGTCATATGATTTGAT
1/gb=AV835569-          TTGGCTAATGTACAAGGTGCGTGATGGCCAAACTTGTGAGTTGGAGTCATATGATTTGAT
                        ____________________________________________________________
consensus               TTGGCTAATGTACAAGGTGCGTGATGGCCAAACTTGTGAGTTGGAGTCATATGATTTGAT

                            .    :    .    :    .    :    .    :    .    :    .    :
12/gb=AK370136+         GTCAGCTAATGATGCTCCCGAGAGGGATCCAATGGACTGGGTCCTTGAAGGGAGCACAGG
9/gb=FD519350-          GTCAGCTAATGATGCTCCCGAGAGGGATCCAATGGACTGGGTCCTTGAAGGGAGCACAGG
8/gb=BJ485214-          GTCAGCTAATGATGCTCCCGAGAGGGATCCAATGGACTGGGTCCTTGAAGGGAGCACAGG
1/gb=AV835569-          GTCAGCTAATGATGCTCCCGAGAGGGATCCAATGGACTGGGTCCTTGAAGGGAGCACAGG
11/gb=GH218900-                                 GGATCCAATGGACTGGGTCCTTGAAGGGAGCACAGG
                        ____________________________________________________________
consensus               GTCAGCTAATGATGCTCCCGAGAGGGATCCAATGGACTGGGTCCTTGAAGGGAGCACAGG

                            .    :    .    :    .    :    .    :    .    :    .    :
12/gb=AK370136+         CCAAGGGTCTACCTGGAATACTATTGATGCTCGGAGTTCTGTAATCTTCAGCAGCCGTTT
9/gb=FD519350-          CCAAGGGTTTACCTGGAATACTATTGATGCTCGGAGTTTTGTAATTTTCAGCAGCCGTTT
8/gb=BJ485214-          CCAAGGGTCTACCTGGAATACTATTGATGCTCGGAGTTCTGTAATCTTCAGCAGCCGTTT
1/gb=AV835569-          CCAAGGGTCTACCTGGAATACTATTGATGCTCGGAGTTCTGTAATCTTCAGCAGCCGTTT
11/gb=GH218900-         CCAAGGGTCTACCTGGAATACTATTGATGCTCGGAGTTCTGTAATCTTCAGCAGCCGTTT
                        ____________________________________________________________
consensus               CCAAGGGTCTACCTGGAATACTATTGATGCTCGGAGTTCTGTAATCTTCAGCAGCCGTTT

                            .    :    .    :    .    :    .    :    .    :    .    :
12/gb=AK370136+         CTGTAGGAAATCATTTACCGTTGACAAGAGACGGAAGGCAAATGTACTTCGGTTTCGGTT
9/gb=FD519350-          TTGTAGGAAATCATTTACCGTTGACAAGAGACGGAAGGCAAATGTACTTCGGTTTCGGTT
8/gb=BJ485214-          CTGTAGGAAATCATTTACCGTTGACAAGAGACGGAAGGCAAATGTACTTCGGTTTCGGTT
1/gb=AV835569-          CTGTAGGAAATCATTTACCGTTGACAAGAGACGGAAGGCAAATGTACTTCGGTTTCGGTT
11/gb=GH218900-         CTGTAGGAAATCATTTACCGTTGACAAGAGACGGAAGGCAAATGTACTTCGGTTTCGGTT
                        ____________________________________________________________
consensus               CTGTAGGAAATCATTTACCGTTGACAAGAGACGGAAGGCAAATGTACTTCGGTTTCGGTT

                            .    :    .    :    .    :    .    :    .    :    .    :
12/gb=AK370136+         TCTACGTGTGAGAGAATCTAGTGCCAATCCTAGGTTTCAAATAGGATCCATCGACTTTTA
9/gb=FD519350-          TTTACGTGTGAGAGAATTTAGTGCCAATCCTAGGTTTCAAATAGGATCCATCGACTTTTA
8/gb=BJ485214-          TCTACGTGTGAGAGAATCTAGTGCCAATCCTAGGTTTCAAATAGGATCCATCGACTTCTA
1/gb=AV835569-          TCTACGTGTGAGAGAATCTAGTGCCAATCCTAGGTTTCAAATAGGATCCATCGACTTCTA
11/gb=GH218900-         TCTACGTGTGAGAGAATATAGTGCCAATCCTAGGTTTCAAATAGGATCCATCGACTTCTA
                        ____________________________________________________________
consensus               TCTACGTGTGAGAGAATCTAGTGCCAATCCTAGGTTTCAAATAGGATCCATCGACTTCTA

                            .    :    .    :    .    :    .    :    .    :    .    :
12/gb=AK370136+         CGGAAAAAACCACATGGCCTGATCGCAGGTGCTAATTTGTGTCTACAAAACCCAACAAAA
9/gb=FD519350-          CGGAAAAAACC
8/gb=BJ485214-          CGGAAAAAACCACATGGCCTGATCGCAGGTGCTAATTTGTGTCTACAAAACCCAACAAAA
1/gb=AV835569-          CGGAAAAAACCACATGGCCTGATCGCAGGTGCTAATTTGTGTCTACAAAACCCAACAAAA
11/gb=GH218900-         CGGAAAAAACCACATGGCCTGATCGCAGGTGCTAATTTGTGTCTACAAAACCCAACAAAA
                        ____________________________________________________________
consensus               CGGAAAAAACCACATGGCCTGATCGCAGGTGCTAATTTGTGTCTACAAAACCCAACAAAA

                            .    :    .    :    .    :    .    :    .    :    .    :
12/gb=AK370136+         CCTTGTCGAATAGTGCAGCTATCATGGCCAGTATGTGCAACCAATAATTGTACTATTTAT
8/gb=BJ485214-          CCTTGTCGAATAGTGCAGCTATCATGGCCAGTATGTGCAACCAATAATTGTACNATTTAT
1/gb=AV835569-          CCTTGTCGAATAGTGCAGCTATCATGGCCAGTATGTGCAACCAATAATTGTACTATTTAT
11/gb=GH218900-         CCTTGTCGAATAGTGCAGCTATCATGGCCAGTATGTGCAACCAATAATTGTACTATTTAT
                        ____________________________________________________________
consensus               CCTTGTCGAATAGTGCAGCTATCATGGCCAGTATGTGCAACCAATAATTGTACTATTTAT

                            .    :    .    :    .    :    .    :    .    :    .    :
12/gb=AK370136+         CTTGGATGATTATAAATTCCGGTTCTACTC
11/gb=GH218900-         GTTGGATGATTATAAATCCCGGTTCTACTCAAAAAAAAAAAAAAAAA
                        ____________________________________________________________
consensus               CTTGGATGATTATAAATCCCGGTTCTACTCAAAAAAAAAAAAAAAAA
```

Figure S2. Catalytic transglutaminase core in plant proteins closely related to AtPng1

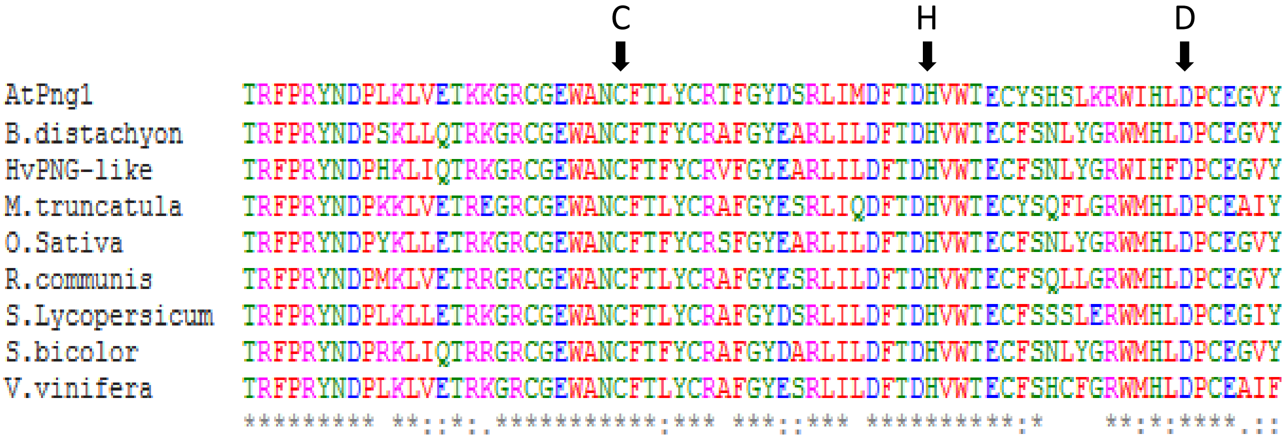

Figure S3. CAP3 assembly of sequences from the UniGene Hv.1565. Sequence AK361332 is a cDNA corresponding to HvTGZ21-like protein, acc. no BAJ92539.1. Coding sequence has been highlighted.

```
******************* Contig 1 ********************
gb=AK361332+
                    gb=CA004201+ is in gb=AK361332+
                    gb=BY860982- is in gb=AK361332+
                    gb=BY839165+ is in gb=AK361332+
                    gb=BG366894+ is in gb=AK361332+
                    gb=EX578617- is in gb=AK361332+
                    gb=CB859768- is in gb=AK361332+
                    gb=BY873374- is in gb=CB859768-
                    gb=BY866443- is in gb=CB859768-
                    gb=EX578618+ is in gb=AK361332+
                    gb=AL501623- is in gb=EX578618+
                    gb=BU998121+ is in gb=AK361332+
                    gb=BE413043+ is in gb=AK361332+
                    gb=AL504293+ is in gb=AK361332+
                    gb=AJ484188+ is in gb=AL504293+
                    gb=BU998587+ is in gb=AK361332+


DETAILED DISPLAY OF CONTIGS
******************* Contig 1 ********************
                          .    :    .    :    .    :    .    :    .    :    .    :
gb=AK361332+          AAAAAGCGAGCCTCCCTCCCGAGCGCGACAGCTAGGGTTCCTAGCCGCCGCCACCACTG-
gb=CA004201+                              GAGCGCGACAGCTAGGGTTCCTAGCCGCCGCCACCACTGA
gb=BU998121+                                       AGCTAGGGTTCCTAGCCGCCGCC-CCACTG-
                      ____________________________________________________________
consensus             AAAAAGCGAGCCTCCCTCCCGAGCGCGACAGCTAGGGTTCCTAGCCGCCGCCACCACTG-

                          .    :    .    :    .    :    .    :    .    :    .    :
gb=AK361332+          CCGGCCCGCCGCCGCGGCGCGCCCAGCCGGCCGGAGCCTCCGCTGCCCCGCCTTCGGTAC
gb=CA004201+          CCGGCCCGCCGCCGCGGCGCGCCCAGCCGGCCGGAGCCTCCGCTGCCCACGCTTCGGTAC
gb=BU998121+          CCGGCCCGCCGCCGCGGCGCGCCCAGCCGGCCGGAGCCTCCGCTGCCCCGCCTTCGGTAC
                      ____________________________________________________________
consensus             CCGGCCCGCCGCCGCGGCGCGCCCAGCCGGCCGGAGCCTCCGCTGCCCCGCCTTCGGTAC

                          .    :    .    :    .    :    .    :    .    :    .    :
gb=AK361332+          AATATCCCAGCAGCGCATCCCTCCGTCAAGCAGCCGCTCCCACCAGCGGCGCCTCCCTCC
gb=CA004201+          AATATCCCAGCAGCGCATCCCTCCGTCAAGCAGCCGCTCCCACCAGCGGCGCCTCCCTCC
gb=BU998121+          AATATCCCAGCAGCGCATCCCTCCGTCAAGCAGCCGCTCCCACCAGCGGCGCCTCCCTCC
                      ____________________________________________________________
consensus             AATATCCCAGCAGCGCATCCCTCCGTCAAGCAGCCGCTCCCACCAGCGGCGCCTCCCTCC

                          .    :    .    :    .    :    .    :    .    :    .    :
gb=AK361332+          CTGGAGCAGGCGCTCCCGCCCGGAGCTACGCCGGCGACCAGTCCATAGGCAGGGCATCTA
gb=CA004201+          CTGGAGCAGGCGCTCCCGCCCGGAGCTACGCCGGCGACCAGTCCATAGGCAGGGCATCTA
gb=BU998121+          CTGGAGCAGGCGCTCCCGCCCGGAGCTACGCCGGCGACCAGTCCATAGGCAGGGCATCTA
                      ____________________________________________________________
consensus             CTGGAGCAGGCGCTCCCGCCCGGAGCTACGCCGGCGACCAGTCCATAGGCAGGGCATCTA

                          .    :    .    :    .    :    .    :    .    :    .    :
gb=AK361332+          CATTGTCAATTGGAGGGGCTTGGAACTGCTTCGGCATCAACAGTAGAGCAAACACGAAGG
gb=CA004201+          CATTGTCAATTGGAGGGGCTTGGAACTGCTTCGGCATCAACAGTAGAGC
gb=BU998121+          CATTGTCAATTGGAGGGGCTTGGAACTGCTTCGGCATCAACAGTAGAGCAAACACGAAGG
```

```
gb=BU998587+                                     AACAGTAGAGCAAACACGAAGG
                        ____________________________________________________________
consensus               CATTGTCAATTGGAGGGGCTTGGAACTGCTTCGGCATCAACAGTAGAGCAAACACGAAGG

                            .    :    .    :    .    :    .    :    .    :    .    :
gb=AK361332+            ATAATGGCTCATCGTGGACACCTAGATGGACTAATGCGCCATGGTGCATTCTCTGGAGCC
gb=BU998121+            ATAATGGCTCATCGTGGACACCTAGATGGACTAATGCGCCATGGTGCATTCTCTGGAGCC
gb=BE413043+                         GTGGACACCTACATGGACTAATGCGCCATGGGGCATTCTCTGGAGCC
gb=AL504293+                         GTGGACACCTACATGGACTAATGCGCCATGGGGCATTCTCTGGAGCC
gb=BU998587+            ATAATGGCTCATCGTGGACACCTAGATGGACTAATGCGCCATGGTGCATTCTCTGGAGCC
                        ____________________________________________________________
consensus               ATAATGGCTCATCGTGGACACCTAGATGGACTAATGCGCCATGGTGCATTCTCTGGAGCC

                            .    :    .    :    .    :    .    :    .    :    .    :
gb=AK361332+            GGCCTCTCTGGTCGCCAACCTTTGGAGCCTTCTCCTGCCACCATACTGGAGATCCTGGAA
gb=BU998121+            GGCCTCTCTGGTCGCCAACCTTTGGAGCCTTCTCCTGCCACCATACTGGAGATCCTGGAA
gb=BE413043+            GGCCTCTCTGGTCGCCAACCTTTGGAGCCTTCTCCTGCCACCATACTGGAGATCCTGGAA
gb=AL504293+            GGCCTCTCTGGTCGCCAACCTTTGGAGCCTTCTCCTGCCACCATACTGGAGATCCTGGAA
gb=BU998587+            GGCCTCTCTGGTCGCCAACCTTTGGAGCCTTCTCCTGCCACCATACTGGAGATCCTGGAA
                        ____________________________________________________________
consensus               GGCCTCTCTGGTCGCCAACCTTTGGAGCCTTCTCCTGCCACCATACTGGAGATCCTGGAA

                            .    :    .    :    .    :    .    :    .    :    .    :
gb=AK361332+            AACAAGCTTGCCGTGCAGACCGCAGAGGCAGAAAAACTTATCAGAGAAAATCAGCGATTG
gb=BU998121+            AACAAGCTTGCCGTGCAGACCGCAGAGGCAGAAAAACTTATCAGAGAAAATCAGCGATTG
gb=BE413043+            AACAAGCTTGCCGTGCAGACCGCAGAGGCAGAAAAACTTATCAGAGAAAATCAGCGATTG
gb=AL504293+            AACAAGCTTGCCGTGCAGACCGCAGAGGCAGAAAAACTTATCAGAGAAAATCAGCGATTG
gb=AJ484188+                                                CTTATCAGAGAAAATCAGCGATTG
gb=BU998587+            AACAAGCTTGCCGTGCAGACCGCAGAGGCAGAAAAACTTATCAGAGAAAATCAGCGATTG
                        ____________________________________________________________
consensus               AACAAGCTTGCCGTGCAGACCGCAGAGGCAGAAAAACTTATCAGAGAAAATCAGCGATTG

                            .    :    .    :    .    :    .    :    .    :    .    :
gb=AK361332+            GCAGATAGCCATGCGGCCTTGAGAAAGGATATTATTGGCACTGAGACAGAAATGCAAATG
gb=BY839165+                                                TATTGGCACTGAGACAGAAATGCAAATG
gb=BU998121+            GCAGATAGCCATGCGGCCTTGAGAAAGGATATTATTGGCACTGAGACAGAAATGCAAATG
gb=BE413043+            GCAGATAGCCATGCGGCCTTGAGAAAGGATATTATTGGCACTGAGACAGAAATGCAAATG
gb=AL504293+            GCAGATAGCCATGCGGCCTTGAGAAAGGATATTATTGGCACTGAGACAGAAATGCAAATG
gb=AJ484188+            GCAGATAGCCATGCGGCCTTGAGAAAGGATATTATTGGCACTGAGACAGAAATGCAAATG
gb=BU998587+            GCAGATAGCCATGCGGCCTTGAGAAAGGATATTATTGGCACTGAGACAGAAATGCAAATG
                        ____________________________________________________________
consensus               GCAGATAGCCATGCGGCCTTGAGAAAGGATATTATTGGCACTGAGACAGAAATGCAAATG

                            .    :    .    :    .    :    .    :    .    :    .    :
gb=AK361332+            ATTCGCACCCACCTAGGTGATGTCCAGGTAGAGACTGATATGCACATGAGAGATTTGGTG
gb=BY839165+            ATTCGCACCCACCTAGGTGATGTCCAGGTAGAGACTGATATGCACATGAGAGATTTGGTG
gb=BU998121+            ATTCGCACCCACCTAGGTGATGTCCAGGTAGAGACTGATATGCACATGAGAGATTTGGTG
gb=BE413043+            ATTCGCACCCACCTAGGTGATGTCCAGGTAGAGACTGATATGCACATGAGAGATTTGGTG
gb=AL504293+            ATTCGCACCCACCTAGGTGATGTCCAGGTAGAGACTGATATGCACATGAGAGATTTGGTG
gb=AJ484188+            ATTCGCACCCACCTAGGTGATGTCCAGGTAGAGACTGATATGCACATGATAGATTTGGTG
gb=BU998587+            ATTCGCACCCACCTAGGTGATGTCCAGGTAGAGACTGATATGCACATGAGAGATTTGGTG
                        ____________________________________________________________
consensus               ATTCGCACCCACCTAGGTGATGTCCAGGTAGAGACTGATATGCACATGAGAGATTTGGTG

                            .    :    .    :    .    :    .    :    .    :    .    :
gb=AK361332+            GAGAGAATCAGATTAATGGAGGCGGACATACGGGCTGGTGAGGCAGTGATGAAGGAACTT
gb=BY839165+            GAGAGAATCAGATTAATGGAGGCGGACATACGGGCTGGTGAGGCAGTGATGAAGGAACTT
gb=BU998121+            GAGAGAATCAGATTAATGGAGGCGGACATACGGGCTGGTGAGGCAGTGAT
gb=BE413043+            GAGAGAATCAGATTAATGGAGGCGGACATACGGGCTGGTGAGGCAGTGATGAAGGAACTT
gb=AL504293+            GAGAGAATCAGATTAATGGAGGCGGACATACGGGCTGGTGAGGCAGTGATGAAGGAACTT
gb=AJ484188+            GAGAGAATCAGATTAATGGAGGCGGACATACGGGCT
gb=BU998587+            GAGAGAATCAGATTAATGGAGGCGGACATACGGGCTGGTGAGGCAGTGATGAAGGAACTT
                        ____________________________________________________________
consensus               GAGAGAATCAGATTAATGGAGGCGGACATACGGGCTGGTGAGGCAGTGATGAAGGAACTT

                            .    :    .    :    .    :    .    :    .    :    .    :
gb=AK361332+            CACCAAGTGCATATGGAGGCAAAGCGACTTATTGCTGAAAGGCAGAGGCTTACTAATGAT
gb=BY839165+            CACCAAGTGCATATGGAGGCAAAGCGACTTATTGCTGAAAGGCAGAGGCTTACTAATGAT
gb=BE413043+            CACCAAGTGCATATGGAGGCAAAGCGACTTATTGCTGAAAGGCAGAGGCTTACTAATGAT
gb=AL504293+            CACCAAGTGCATATGGAGGCAAAGCGACTTATTGCTGAAAGGCAGAGGCTTACTAATGAT
gb=BU998587+            CACCAAGTGCATATGGAGGCAAAGCGACTTATTGCTGAAAGGCAGAGGCTTACTAATGAT
                        ____________________________________________________________
consensus               CACCAAGTGCATATGGAGGCAAAGCGACTTATTGCTGAAAGGCAGAGGCTTACTAATGAT

                            .    :    .    :    .    :    .    :    .    :    .    :
gb=AK361332+            GTAGAGGCTGCGACTAAAGAACTACAGAAGTACTCTGGTGACAATAGTAACCTTGCTGAA
```

```
gb=BY839165+          GTAGAGGCTGCGACTAAAGAACTACAGAAGTACTCTGGTGACAATAGTAACCTTGCTGAA
gb=BE413043+          GTANAGGCTGCGACTAAAGAACTACAGAAGTACTCTGGTGACAATAGTAACCTTGCTGAA
gb=AL504293+          GTANAGGCTGCGACTAAAGAACTACAGAAGTACTCTGGTGACAATAGTAACCTTGCTGAA
gb=BU998587+          GTAGAGGCTGCGACTAAAGAACTACAGAAGTACTCTGGTGACAATAGTAACCTTGCTGAA
                      ____________________________________________________________
consensus             GTAGAGGCTGCGACTAAAGAACTACAGAAGTACTCTGGTGACAATAGTAACCTTGCTGAA

                               .         :         .         :         .         :
gb=AK361332+          TTGGTTGCGGAGCTAGATGGTCTACGGAAAGAGCATCATGGTCTAAGATCTGCCTTCGAG
gb=BY839165+          TTGGTTGCGGAGCTAGATGGTCTACGGAAAGAGCATCATGGTCTAAGATCTGCCTTCGAG
gb=BG366894+                                      AAGAGCATCACGGTCTAAGATCTGCCTTCGAG
gb=BE413043+          TTGGTTGCGGAGCTAGATGGTCTACGGAAAGAGCATCATGGTCTAAGATCTGCCTTCGAG
gb=AL504293+          TTGGTTGCGGAGCTAGATGGTCTACGGAAAGAGCATCATGGTCTAAGATCTGCCTTCGAG
gb=BU998587+          TTGGTTGCGGAGCTAGATGGTCTACGGAAAGAGCATCATGGTCTAAGATCTGCCTTCGAG
                      ____________________________________________________________
consensus             TTGGTTGCGGAGCTAGATGGTCTACGGAAAGAGCATCATGGTCTAAGATCTGCCTTCGAG

                               .         :         .         :         .         :
gb=AK361332+          TATGAGAAAAACACAAACATCAAGCAAGTT-GAGCAGATGCGGACCATGGAAATGAACTT
gb=BY839165+          TATGAGAAAAACACAAACATCAAGCAAGTT-GAGCAGATGCGGACCCATGGAATGAACTT
gb=BG366894+          TATGAGAAAAACACAAACATCAAGCAAGTT-GAGCAGATGCGGACCATGGAAATGAACTT
gb=BE413043+          TATGAGAAAAACACAAACATCAAGCANGTTTGAGCAGATGCGGACCATGGAAATGAACTT
gb=AL504293+          TATGAGAAAAACACAAACATCAAGCANGTTTGAGCAGATGCGGACCATGGAAATGAACTT
gb=BU998587+          TATGAGAAAAACACAAACATCAAGCAAGTT-GAGCAGATGCGGACCATGGAAATGAACTT
                      ____________________________________________________________
consensus             TATGAGAAAAACACAAACATCAAGCAAGTT-GAGCAGATGCGGACCATGGAAATGAACTT

                               .         :         .         :         .         :
gb=AK361332+          GATAACCATGACTAAAGAGGCGGACAAGTTACGAGCTGATTTGGCAAATGCTGCAAACCG
gb=BY839165+          GATAACCATGACTAAAGAGGCGGANCAGTTACGAGCTGATTTGGCAAATGCTGCAAACCG
gb=BG366894+          GATAACCATGACTAAAGAGGCGGACAAGTTACGAGCTGATTTGGCAAATGCTGCAAACCG
gb=BE413043+          GATAACCATGACTAAAGAGGCGGACATGTTACGAGCTGATTTGGCAAATGCTGCAAACCG
gb=AL504293+          GATAACCATGACTAAAGAGGCGGACATGTTACGAGCTGATTTGGCAAATGCTGCAAACCG
gb=BU998587+          GATAACCATGACTAAAGAGGCGGACAAGTTACGAGCTG
                      ____________________________________________________________
consensus             GATAACCATGACTAAAGAGGCGGACAAGTTACGAGCTGATTTGGCAAATGCTGCAAACCG

                               .         :         .         :         .         :
gb=AK361332+          AGCACATGCAGCACAGGTTACACC-TCCACAGCCTGGGACAGCACAAGCTGCAGCGGCTT
gb=BY839165+          AGCACATGCAGCACAGGTTACACC
gb=BG366894+          AGCACATGCAGCACAGGTTACACC-TCCACAGCCTGGGACAGCACAAGCTGCAGCGGCTT
gb=BE413043+          AGCACATGCAGCACAGGTTACACCCTCCACANCCTGGGACAGCACAAGCTGCAGCGGCTT
gb=AL504293+          AGCACATGCAGCACAGGTTACACC
                      ____________________________________________________________
consensus             AGCACATGCAGCACAGGTTACACC-TCCACAGCCTGGGACAGCACAAGCTGCAGCGGCTT

                               .         :         .         :         .         :
gb=AK361332+          CAGCAGCCACAAATCCATATGCAAGTGCATATACCAATCACCCCTCCGCATATCAGCAAG
gb=BG366894+          CAGCAGCCACAAATCCATATGCAAGTGCATATACCAATCACCCCTCCGCATATCAGCAAG
gb=BE413043+          CAGCAGC
                      ____________________________________________________________
consensus             CAGCAGCCACAAATCCATATGCAAGTGCATATACCAATCACCCCTCCGCATATCAGCAAG

                               .         :         .         :         .         :
gb=AK361332+          GAACTTCCCAAGCCACGGCATACCAGCAAGGAATCCCCCAAGCCGCGGCATACCAGCAAG
gb=BG366894+          GAACTTCCCAAGCCGCGGCATACCAGCAAGGAATCCCCCAAGCCGCGGCATACCAGCAAG
                      ____________________________________________________________
consensus             GAACTTCCCAAGCCACGGCATACCAGCAAGGAATCCCCCAAGCCGCGGCATACCAGCAAG

                               .         :         .         :         .         :
gb=AK361332+          GAACTTCCCAAGCCACGGCATACCAGCAAGGAACTTCCCAAGCCGCGGCATACCAGCAAG
gb=BG366894+          GAACTTCCCAAGCCACGGCATACCAGCAAGGAACTTCCCAAGCCGCGGCATACCAGCAAG
                      ____________________________________________________________
consensus             GAACTTCCCAAGCCACGGCATACCAGCAAGGAACTTCCCAAGCCGCGGCATACCAGCAAG

                               .         :         .         :         .         :
gb=AK361332+          GAACTTCCCAAGCCGCGGCATACCAGCAAGGAACCCCCCAAGCCGCGGCATACCAGCAAG
gb=BG366894+          GAACTTCCCAAGCCGCGGCATTACCAGCAGGAACCCCCCAAGCCGCGGCATACCAGCAAG
                      ____________________________________________________________
consensus             GAACTTCCCAAGCCGCGGCATAACAACAAGGAACCCCCCAAGCCGCGGCATACCAGCAAG

                               .         :         .         :         .         :
gb=AK361332+          GAACCCCCCAAGCCGCGGCATACCAGCAAGGAGGACACCCCCAAGCCGCGGCATACCAGC
gb=BG366894+          GAACCCCTCAAGCCGCGGCATACCAGCAAGGA
                      ____________________________________________________________
consensus             GAACCCCCCAAGCCGCGGCATACCAGCAAGGAGGACACCCCCAAGCCGCGGCATACCAGC
```

```
                          .    :    .    :    .    :    .    :    .    :    .    :
gb=AK361332+          AAGGAGGACACCCCCAAGCTGGATCATACCAACAAGGACCTCCCCAAGCTGGAGCATATC
gb=CB859768-                                                             GCATATC

consensus             AAGGAGGACACCCCCAAGCTGGATCATACCAACAAGGACCTCCCCAAGCTGGAGCATATC

                          .    :    .    :    .    :    .    :    .    :    .    :
gb=AK361332+          AACAAGGACCTCCCCAAGGGGCATACGCTTACCCAACAGCTTATGATTCTGCCACCGCTT
gb=EX578617-                                              CAGCTTATGATTCTGCCACCGCTT
gb=CB859768-          AACAAGGACCTCCCCAAGGGGCATACGCTTACCCAACAGCTTATGATTCTGCCACCGCTT
gb=BY873374-                                                             ACCGCTT
gb=BY866443-                                                                   T
gb=EX578618+               GGACCTCCCCAAGGGGCATACGCTTACCCAACAGCTTATGATTCTGCCACCGCTT

consensus             AACAAGGACCTCCCCAAGGGGCATACGCTTACCCAACAGCTTATGATTCTGCCACCGCTT

                          .    :    .    :    .    :    .    :    .    :    .    :
gb=AK361332+          ACCAGCTGCAGGCTAATGCGTATGCTAGCTATTCT-GGCTATCCAGTTGCAGGTTATGCG
gb=EX578617-          ACCAGCTGCAGGCTAATGCGTATGCTAGCTATTCT-GGCTATCCAGTTGCAGGTTATGCG
gb=CB859768-          ACCAGCTGCAGGCTAATGCGTATGCTAGCTATTCT-GGCTATCCAGTTGCAGGTTATGCG
gb=BY873374-          ACCAGCTGCAGGCTAATGCGTATGCTAGCTATTCT-GGCTATCCAGTTGCAGGTTATGCG
gb=BY866443-          ACCAGCTGCAGGCTAATGCGTATGCTAGCTATTCT-GGCTATCCAGTTGCAGGTTATGCG
gb=EX578618+          ACCAGCTGCAGGCTAATGCGTATGCTAGCTATTCT-GGCTATCCAGTTGCAGGTTATGCG
gb=AL501623-                        AATGCGTATGCTAGCTATTCTTGGCTANCCAGTTGCAGGTTATGCG

consensus             ACCAGCTGCAGGCTAATGCGTATGCTAGCTATTCT-GGCTATCCAGTTGCAGGTTATGCG

                          .    :    .    :    .    :    .    :    .    :    .    :
gb=AK361332+          CAACCCAACTATCCTGGCACGTACGCTGCACCTCAGCACCCAGTAGCCAGTGGCGCGGCT
gb=BY860982-                       CTGGCACGTACGCTGCACCTCAGCACCCAGTAGCCAGTGGCGCGGCT
gb=EX578617-          CAACCCAACTATCCTGGCACGTACGCTGCACCTCAGCACCCAGTAGCCAGTGGCGCGGCT
gb=CB859768-          CAACCCAACTATCCTGGCACGTACGCTGCACCTCAGCACCCAGTAGCCAGTGGCGCGGCT
gb=BY873374-          CAACCCAACTATCCTGGCACGTACGCTGCACCTCAGCACCCAGTAGCCAGTGGCGCGGCT
gb=BY866443-          CAACCCAACTATCCTGGCACGTACGCTGCACCTCAGCACCCAGTAGCCAGTGGCGCGGCT
gb=EX578618+          CAACCCAACTATCCTGGCACGTACGCTGCACCTCAGCACCCAGTAGCCAGTGGCGCGGCT
gb=AL501623-          CAACCCAACTATCCTGGCACGTACGNTGCACCTCAGCACCCAGTAGCCAGTGGCGCGGCT

consensus             CAACCCAACTATCCTGGCACGTACGCTGCACCTCAGCACCCAGTAGCCAGTGGCGCGGCT

                          .    :    .    :    .    :    .    :    .    :    .    :
gb=AK361332+          ACTGATGCCACGAGCGCGTACGGTGCGGCTGGTAGCACGGGATATCCTGCTGCACCGGTT
gb=BY860982-          ACTGATGCCACGAGCGCGTACGGTGCGGCTGGTAGCACGGGATATCCTGCTGCACCGGTT
gb=EX578617-          ACTGATGCCACGAGCGCGTACGGTGCGGCTGGTAGCACGGGATATCCTGCTGCACCGGTT
gb=CB859768-          ACTGATGCCACGAGCGCGTACGGTGCGGCTGGTAGCACGGGATATCCTGCTGCACCGGTT
gb=BY873374-          ACTGATGCCACGAGCGCGTACGGTGCGGCTGGTAGCACGGGACATCCTGCTGCACCGGTT
gb=BY866443-          ACTGATGCCACGAGCGCGTACGGTGCGGCTGGTAGCACGGGATATCCTGCTGCACCGGTT
gb=EX578618+          ACTGATGCCACGAGCGCGTACGGTGCGGCTGGTAGCACGGGATATCCTGCTGCACCGGTT
gb=AL501623-          ACTGATGCCACGAGCGCGTACGGTGCGGCTGGTAGCACGGGATATCCTGCTGCACCGGTT

consensus             ACTGATGCCACGAGCGCGTACGGTGCGGCTGGTAGCACGGGATATCCTGCTGCACCGGTT

                          .    :    .    :    .    :    .    :    .    :    .    :
gb=AK361332+          CAGGCAAGCAGCGGGGCTGCCAATGCGGGGCAAGCACCGCCAGCTCCTTATCCTGCAACG
gb=BY860982-          CAGGCAAGCAGCGGGGCTGCCAATGCGGGGCAAGCACCGCCAGCTCCTTATCCTGCAACG
gb=EX578617-          CAGGCAAGCAGCGGGGCTGCCAATGCGGGGCAAGCACCGCCAGCTCCTTATCCTGCAACG
gb=CB859768-          CAGGCAAGCAGCGGGGCTGCCAATGCGGGGCAAGCACCGCCAGCTCCTTATCCTGCAACG
gb=BY873374-          CAGGCAAGCAGCGGGGCTGCCAATGCGGGGCAAGCACCGCCAGCTCCTTATCCTGCAACG
gb=BY866443-          CAGGCAAGCAGCGGGGCTGCCAATGCGGGGCAAGCACCGCCAGCTCCTTATCCTGCAACG
gb=EX578618+          CAGGCAAGCAGCGGGGCTGCCAATGCGGGGCAAGCACCGCCAGCTCCTTATCCTGCAACG
gb=AL501623-          CAGGCAAGCAGCGGGGCTGCCAATGCGGGGCAAGCACCGCCAGCTCCTTATCCTGCAACG

consensus             CAGGCAAGCAGCGGGGCTGCCAATGCGGGGCAAGCACCGCCAGCTCCTTATCCTGCAACG

                          .    :    .    :    .    :    .    :    .    :    .    :
gb=AK361332+          TATGACCCAACCAAAGCAGCCCAGAGGTGAAAAATCCGGGGTCGTAACCTGAAGATGGCC
gb=BY860982-          TATGACCCAACCAAAGCAGCCCAGAGGTGAAAAATCCGGGGTCGTAACCTGAAGATGGCC
gb=EX578617-          TATGACCCAACCAAAGCAGCCCAGAGGTGAAAAATCCGGGGTCGTAACCTGAAGATGGCC
gb=CB859768-          TATGACCCAACCAAAGCAGCCCAGAGGTGAAAAATCCGGGGTCGTAACCTGAAGATGGCC
gb=BY873374-          TATGACCCAACCAAAGCAGCCCAGAGGTGAAAAATCCGGGGTCGTAACCTGAAGATGGCC
gb=BY866443-          TATGACCCAACCAAAGCAGCCCAGAGGTGAAAAATCCGGGGTCGTAACCTGAAGATGGCC
gb=EX578618+          TATGACCCAACCAAAGCAGCCCAGAGGTGAAAAATCCGGGGTCGTAACCTGAAGATGGCC
gb=AL501623-          TATGACCCAACCAAAGCAGCCCAGAGGTGAAAAATCCGGGGTCGTAACCTGAAGATGGCC

consensus             TATGACCCAACCAAAGCAGCCCAGAGGTGAAAAATCCGGGGTCGTAACCTGAAGATGGCC
```

```
                          .    :    .    :    .    :    .    :    .    :    .    :
gb=AK361332+          TGTATGCCCGCAAAATTTTATCCAAAAATTCTCATGGCTCTCATTTCCTTCATGTACATT
gb=BY860982-          TGTATGCCCGCAAAATTTTATCCAAAAATTCTCATGGCTCTCATTTCCTTCATGTACATT
gb=EX578617-          TGTATGCCCGCAAAATTTTATCCAAAAATTCTCATGGCTCTCATTTCCTTCATGTACATT
gb=CB859768-          TGTATGCCCGCAAAATTTTATCCAAAAATTCTCATGGCTCTCATTTCCTTCATGTACATT
gb=BY873374-          TGTATGCCCGCAAAATTTTATCCAAAAATTCTCATGGCTCTCATTTCCTTCATGTACATT
gb=BY866443-          TGTATGCCCGCAAAATTTTATCCAAAAATTCTCATGGCTCTCATTTCCTTCATGTACATT
gb=EX578618+          TGTATGCCCGCAAAATTTTATCCAAAAATTCTCATGGCTCTCATTTCCTTCATGTACATT
gb=AL501623-          TGTATGCCCGCAAAATTTTATCCAAAAATTCTCATGGCTCTCATTTCCTTCATGTACATT
                      ____________________________________________________________
consensus             TGTATGCCCGCAAAATTTTATCCAAAAATTCTCATGGCTCTCATTTCCTTCATGTACATT

                          .    :    .    :    .    :    .    :    .    :    .    :
gb=AK361332+          CATTCACTCCCTGTAGCTTTATGCACACCATGGGGCCGCTGAGAGTCGCAGTTTATGGTC
gb=BY860982-          CATTCACTCCCTGTAGCTTTATGCACACCATGGGGCCGCTGAGAGTCGCAGTTTATGGTC
gb=EX578617-          CATTCACTCCCTGTAGCTTTATGCACACCATGGGGCCGCTGAGAGTCGCAGTTTATGGTC
gb=CB859768-          CATTCACTCCCTGTAGCTTTATGCACACCATGGGGCCGCTGAGAGTCGCAGTTTATGGTC
gb=BY873374-          CATTCACTCCCTGTAGCTTTATGCACACCATGGGGCCGCTGAGAGTCGCAGTTTATGGTC
gb=BY866443-          CATTCACTCCCTGTAGCTTTATGCACACCATGGGGCCGCTGAGAGTCGCAGTTTATGGTC
gb=EX578618+          CATTCACTCCCTGTAGCTTTATGCACACCATGGGGCCGCTGAGAGTCGCAGTTTATGGTC
gb=AL501623-          CATTCACTCCCTGTAGCTTTATGCACACCATGGGGCCGCTGAGAGTCGCAGTTTATGGTC
                      ____________________________________________________________
consensus             CATTCACTCCCTGTAGCTTTATGCACACCATGGGGCCGCTGAGAGTCGCAGTTTATGGTC

                          .    :    .    :    .    :    .    :    .    :    .    :
gb=AK361332+          GGAGTCTTGAATTGATTTGTATCTCTTGTTTTGCTGGATGGTTGAACTGATATAGACGTA
gb=BY860982-          GGAGTCTTGAATTGATTTGTATCTCTTGTTTTGCTGGATGGTTGAACTGATATAGACGTA
gb=EX578617-          GGAGTCTTGAATTGATTTGTATCTCTTGTTTTGCTGGATGGTTGAACTGATATAGACGTA
gb=CB859768-          GGAGTCTTGAATTGATTTGTATCTCTTGTTTTGCTGGATGGTTGAACTGATATAGACGTA
gb=BY873374-          GGAGTC
gb=BY866443-          GGAGTCTTGAATTGATTTGTATCTCTTGTTTTGCTGGATGGTTGAACTGATATAGACGT
gb=EX578618+          GGAGTCTTGAATTGATTTGTATCTCTTGTTTTGCTGGATGGTTGAACTGATATAGACGTA
gb=AL501623-          GGAGTCTTGAATTGATTTGTATCTCTTGTTTTGCTGGATGGTTGAACTGATATAGACGTA
                      ____________________________________________________________
consensus             GGAGTCTTGAATTGATTTGTATCTCTTGTTTTGCTGGATGGTTGAACTGATATAGACGTA

                          .    :    .    :    .    :    .    :    .    :    .    :
gb=AK361332+          ACTGTTGGCTAATATCAGGGATGTTAGAATTACTCTTGCTGTTTTGAAATAGTGGGAAAC
gb=BY860982-          ACTGTTGGCTAATATCAGGGATGTTAGAATT
gb=EX578617-          ACTGTTGGCTAATATCAGGGATGTTAGAATT
gb=CB859768-          ACTGTTGGCTAATATCAG
gb=EX578618+          ACTGTTGGCTAATATCAGGGATGTTAGAATT
gb=AL501623-          ACTGTTGGCTAA
                      ____________________________________________________________
consensus             ACTGTTGGCTAATATCAGGGATGTTAGAATTACTCTTGCTGTTTTGAAATAGTGGGAAAC

                          .    :    .    :    .    :    .    :    .    :    .    :
gb=AK361332+          CTAGCTGTGGTGCGAGGCTTTTTACCC
                      ____________________________________________________________
consensus             CTAGCTGTGGTGCGAGGCTTTTTACCC
```

Figure S4. Clustal2W sequence alignment of plant proteins showing partial similarity to maize TGZ21 protein. Significant similarity only to ~220 N-terminal amino acids of the TGZ21/TGZ15 sequences is maintained. Downstream of this region, the protein sequences become more diverse and several subgroups can be easily identified, with the *Poaceae* forming a distinct subgroup. However, the TGZ21 sequence similarity to other *Poaceae* proteins rapidly discontinues after Gly249. Accession IDs of the proteins are: HvTGZ21-like (BAJ92539.1), TGZ21 (CAD32336.1), S.bicolor (XP_002448045.1), B.distachyon (XP_003579974.1), O.sativa (NP_001053092.1), V.vinifera1 (XP_002277975.1), V.vinifera2 (XP_002282805.1), P.trichocarpa (XP_002308428.1), R.communis1 (XP_002528217.1), R.communis2 (XP_002531483.1), A.lyrata (XP_002887107.1), A.thaliana (NP_176888.2), G.max1 (XP_003551793.1), G.max2 (XP_003531907.1). Except for barley and maize proteins, all the other sequences are from the NCBI refseq database.

```
G.max2            ----MGSKGRMPPPP-HLRRPHPP--GPAAM--------------PHPLAGPFDLLPPPQ 39
G.max1            ----MGSKGRMPPPP-HLRRPHPQ--DPAAM--------------PHPLVVPFDFLPPPQ 39
V.vinifera2       ----MGSKGRIPP---HLRRPLP---GPGLMHPDSFVPGIRP---PHGVFSPFDMLPPPE 47
R.communis2       ----MGSKGRIPPPH-HLRRPLPG--GGIMHPDPFGPGVHGP---PPGPFPAFDMLPPPE 50
A.lyrata          ----MESKGRIHPSHHHMRRPLPGPGGGIAHPETFGSHAAIPPSAAQGAYPSFNMLPPPE 56
A.thaliana        ----MESKGRIHPSHHHMRRPLPGPGGCIAHPETFGNHGAIPPSAAQGVYPSFNMLPPPE 56
P.trichocarpa     ----MSARRHIRPTLEGRVIQAPGMMRHGPFP---------------AGHHTSEPLSRSD 41
R.communis1       ----MAGKGHNPPGLEGRYLRRPESIRHGLFP---------------PSHRQLDRLPPSD 41
V.vinifera1       ----MAARGHRQPSFEGRAAQAPGMMRHGPFPGSG----------HTVGLRSLETAPHPD 46
B.distachyon      ----MAHRGHLDGRSAQAQGLMRHGAFSAAILS-------------GRQHLEPSPATTLE 43
HvTGZ21-like      ----MAHRGHLDG-------LMRHGAFSGAGLS-------------GRQPLEPSPATILE 36
O.sativa          ----MAYHGQLDGR--QASGLMRDGAFPAASLS-------------GRQPLDR-AATALE 40
S.bicolor         MFRIMAHRGHLDGLTAQAPALMHHGSFAAGKLS-------------SHSPLQS--SSTLE 45
TGZ21             ----MAHRGHLDGLTGQAPALMRHGSFAAGSLS-------------SRSPLQS--SSTLE 41
                      *  : :                                              .  :

G.max2            VMEQKLASQHAEMQRLATENQRLAATHGVLRQELAAAQHELQMLHAHVSALKGEREQQIR 99
G.max1            VMEQKLASQHAEMQRLSTENQRLAATHSVLRQELAAAQHEMQMLHGHVVALKGEREQQIR 99
V.vinifera2       VMEQKLAAQHVDMQRLANENQRLAATHGTLRQELAAAQHELQMLQAQIGVMKSEREQRMR 107
R.communis2       VLEQKLAGQQVEIQRLATENQRLAATHVTLRQELAAAQQELQMLHNDIGVLKAEREQQMR 110
A.lyrata          VMEQKFAAQHGELQRLAIENQRLGATHGSLRQELAAAQHELQMLHSQIGSMKSEREQRMM 116
A.thaliana        VMEQKFVAQHGELQRLAIENQRLGGTHGSLRQELAAAQHEIQMLHAQIGSMKSEREQRMM 116
P.trichocarpa     LLEHRFAAQAADIEQLAGDNNRLVTSHMALREDLAAAQQEVQRLKAHIRSIQTESDIQIR 101
R.communis1       LLENKIAAQAAEIEQLAGDNHRLAATHMALRQDLVDAQQEVKRRKAHIRSIQTESDIQMR 101
V.vinifera1       IAENKMLAQVAEIERLAGDNHRLAATHGALRQELVAAQHEISRIKAQIRSIETESDIQIR 106
B.distachyon      ILEDKLAMLTAEAEKLIRENQRLSSSHQVLRQDIIDTEKEMQMIRTHLGDVQLDTDMQIR 103
HvTGZ21-like      ILENKLAVQTAEAEKLIRENQRLADSHAALRKDIIGTETEMQMIRTHLGDVQVETDMHMR 96
O.sativa          ILEKKLAEQTAEAEKLIRENQRLASSHVVLRQDIVDTEKEMQMIRAHLGDVQTETDMHMR 100
S.bicolor         MLENKLAMQTAEVEKLIMENQRLASSHVVLRQDIVDTEKEMQMIRTHLGEVQTETDLQIR 105
TGZ21             MLENKLAMQTTEVEKLITENQRLASSHVVLRQDIVDTEKEMQMIRTHLGEVQTETDLQIR 101
                  : *.::     : ::*  :*:**  :*  **:::  :: *:.  : .:  :: : : ::

G.max2            VQLEKISKMEAEAKGAESVKMELQQARGEAQNLVVSRDELVSKAQHLTQELQRVH--ADA 157
G.max1            AQMEKIAKMESEAQGSESVKMELQQARGEAQNLVVSRDELVSKAQHLTQELQRVH--ADA 157
V.vinifera2       SLTDKIAKMEAELKAAEPVKLELQQARADAQSLVAARQELISKVQQLTQDLQRSH--SDV 165
R.communis2       GLMDKIAKMETELKAAEPVRSELQQARTEAEKLVVARQELMTKVHQLTQDLHRAH--ADV 168
A.lyrata          GLAEKVAKMETELQKSEAVKLEMQQARAEARSLVVAREELMSKVHQLTQELQKAR--SDV 174
A.thaliana        GLAEKVAKMETELQKSEAVKLEMQQARAEARSLVVAREELMSKVHQLTQELQKSR--SDV 174
P.trichocarpa     VLLDKIAKMEKDIRAGENVKKDLKQAHVEAQNLVKERQELATQIQQASHELQKIH--TDV 159
R.communis1       MLLDKIAKMEAEIRLGDNVKKELRQAHMEAQSLVKAGQDLTTQIQEASKELQKVR--TDV 159
V.vinifera1       VLMERIAKMEGDIRAGEHVKKELQQAHIEAQSLVTARQELTTQVQQATQELQKAN--ADV 164
B.distachyon      DLVERIRLMEADIQAGDAVKKELHQVHMEAKRLITERQMLTSEVEIVTKELQKLS--GNN 161
HvTGZ21-like      DLVERIRLMEADIRAGEAVMKELHQVHMEAKRLIAERQRLTNDVEAATKELQKYS--GDN 154
O.sativa          DLMERMRLMEADIQAGDAVKKELHQVHMEAKRLIAERQMLTVEMDKVTKELHKFS--GDS 158
S.bicolor         DLLERIRLMEADIHSGDAVKKELHQVHMEAKRLITERQMLTLDIENVIKELQKLSASGDG 165
TGZ21             DLLERIRLMEVDIHSGNVVNKELHQMHMEAKRLITERQMLTLEIEDVTKELQKLSASGDN 161
                     :::  ** : : .: *  :::* : :*. *:   : *  . .   ::*::     :

G.max2            VQIPALISELECLRQEYQHCRATFDYEKKLYNDHLESLQVMEKNYVSMSREVEKLRAELT 217
G.max1            VQIPALISEFECLRQEYQHCRATFDYEKNLYNDHLESLQVMEKNYVSMSREVEKLRAELT 217
V.vinifera2       QQIPALMAELESLRQEYQHCRATYDYEKKLYNDHLESLQVMEKNYMTMAREVEKLRAELT 225
R.communis2       QQIPILISELDSLRQEYQRCRVSFDYEKKLFSDHRESLQVMENNYVTMAREVEKLHLELT 228
A.lyrata          QQIPALMSELENLRQEYQQCRATYDYEKKFYNDHLESLQAMEKNYMTMAREVEKLQAQLM 234
A.thaliana        QQIPALMSELENLRQEYQQCRATYDYEKKFYNDHLESLQAMEKNYMTMAREVEKLQAQLM 234
P.trichocarpa     KSIPDLHAELENSRHELKRLRATFEYEKGLNIEKVEQMRAMEQNLIGMAREMENLRVDVL 219
R.communis1       SIIPDLQAELENLRHEYKRLRAMFEYEKGLNIEKVERLQAMEQNLIRMARELEKLHAGVL 219
V.vinifera1       KRLPELHAELDSMGQEHQRLRSTFEYEKGLNVDQVREMQEMEKNLVVMAREVEKLRAEVL 224
B.distachyon      TNLAELLAELDGLRKEHHSLRSAFEYEKNTNVKQVEQMRTMEMNLITMTKEADKLRFDVA 221
HvTGZ21-like      SNLAELVAELDGLRKEHHGLRSAFEYEKNTNIKQVEQMRTMEMNLITMTKEADKLRADLA 214
O.sativa          KKLPELLTELDGLRKEHQSLRSAFEYEKNTNIKQVEQMRTMEMNLMTMTKEADKLRADVA 218
S.bicolor         KSLPELLAELDGLRKEHHNLRSQFEFEKNTNIKQVEQMRTMEMNLITMTKQAEKLRGDVA 225
TGZ21             KSLPELLSELDRLRKEHHNLRSQFEFEKNTNVKQVEQMRTMEMNLITMTKQAEKLRVDVA 221
                    :. * :*::   :* :  *  :::**    .: . :: ** * : *::: ::*:  :

G.max2            NTANVDRR--SSGPYGGTSGTNENEASG-----------LPVGQNAYEDGYSFMQ----- 259
G.max1            NTANVDRR--SSGPYGGTSGTNENEASG-----------LPVGQNAYEDGYSVMQ----- 259
V.vinifera2       NTANMDRR--TGGPYGGPTGYNEGEASGH----------HPIGHNAYEDGYGVPQ----- 268
R.communis2       NTSNVDIRTVTGGPYGGATGNNENETSS-----------RSVGENKYEDSYGVSQSQ--- 274
A.lyrata          NSANSDRR--AGVPYG-SNMNAEIDASG-----------HQSGNGYYEDAFGPQG----- 275
A.thaliana        NNANSDRR--AGGPYG-NNINAEIDASG-----------HQSGNGYYEDAFGPQG----- 275
P.trichocarpa     N---AETRARAPNQYIGGYA--NP------------------------DGYGRPF----- 245
R.communis1       N---AEKKAHAPNLYGGGYVTRNPSCSF-----------PIQRGAASVDSYGRHL----- 260
V.vinifera1       N---AEKRAHAPNPYSGSYP--EPSYPP-----------PTQGSGAYIDSYGRPH----- 263
B.distachyon      NAENR------------AHAAQVTAPQPG-------TAQAAGAS-AATNPYASAYTN--- 258
HvTGZ21-like      NAANR------------AHAAQVTPPQPG-------TAQAAAAS-AATNPYASAYTNHPS 254
O.sativa          NAEKRAQVAAAQAVAAQAGVAHVTASQPG-------AAQAVAVP-AASNPYSSAFTG--- 267
S.bicolor         NAERR--AQAAAQAAAHAAGAQVTASQPG-------TAQATAVSAAATDPYAGAYAS--- 273
TGZ21             NAERR-AQAAAAQAAAHAAGAQVTASQPGQLKLPRFQQQQPQTHMQVHIPATPLHISREP 280
```

Fig S4 Continued.

```
G.max2          ---------------------------GRGPLP-------AASG--------GGGNATTL 277
G.max1          ---------------------------GRGPLP-------AAAGS-------GGGNATTI 278
V.vinifera2     ---------------------------GRAPLPGATGGGVAAAGG-------GGGGGGTP 294
R.communis2     ---------------------------GHTPIPGNSGSAAATAAGSASTGAKAGTGAGTP 307
A.lyrata        ----------------------------YIPQS--------VAGN--------AAGPNSI 291
A.thaliana      ----------------------------YIPQP--------VAGN--------ATGPNSV 291
P.trichocarpa   -------------------------------------------------------VHMGV 250
R.communis1     -------------------------------------------------------VHTRV 265
V.vinifera1     -------------------------------------------------------AQMIA 268
B.distachyon    -----------------QAAAYQQGNPQAVAYQQ------------------QQGTPQAA 283
HvTGZ21-like    AYQQGTSQATAYQQGIPQAAAYQQGTSQATAYQQGTSQAAAYQQGTSQAAAYQQGTPQAA 314
O.sativa        -----------------HPSAYHQG------------------------------ATQAG 280
S.bicolor       -----------------YPSAYQQG-------------------------------AQAA 285
TGZ21           RLGHISRVLRLGYISREPRLGHISREPRLGHISRGARMGHISRGLRLGHISREPRLGHIS 340

G.max2          ITAGGQPGPAPAGTG----------------------------YDAPRGPGYGASAGPA- 308
G.max1          TTAGGQPGPAPAGTG----------------------------YDAPIGPGYGASTGST- 309
V.vinifera2     AYAGPQSGPAASRPG----------------------------YDGPRGPGYDAPRGPG- 325
R.communis2     TYTGAQSGSASTRSA----------------------------YDTPRGPNYEATKGPS- 338
A.lyrata        VGAAQYPYPGVTQPG----------------------------YFPPR-PGYNFPRGP-- 320
A.thaliana      VGAAQYPYQGVTQPG----------------------------YFPQR-PGYNFPRGP-- 320
P.trichocarpa   GPAGEGIIPYNSSNS----------------------------VVSNVGFGGAAMSTTG- 281
R.communis1     GPAVNGTIPYDNGNN----------------------------AAIICGIGGSAVSSAG- 296
V.vinifera1     GTPSAGMIPY----------------------------------------GSVAIPPAS- 287
B.distachyon    AYQHGTPQAAAYQQG----------------------TLQGGAYQQGTPAGTYAYPTA-- 319
HvTGZ21-like    AYQQGTPQAAAYQQGGHPQAAAYQQGGHPQAGSYQQGPPQAGAYQQGPPQGAYAYPTA-- 372
O.sativa        VYQQGTTQAGAYQQG-------------------------------STQAGAYAYPT--- 306
S.bicolor       AYQQG-AQAAAYQLG---------------------------AYQHGTQAGAYQQGN--- 314
TGZ21           REPRLGHISRVLRLGHISRVLRLGYISREPRLGHISREPRLGHISRGARMGHISRGLRLG 400

G.max2          ------------YDAQRVATYDAQRLTGYDAFRGSAYDSKRGPIFDAQRT---------- 346
G.max1          ------------YDAQRGATYDTQRLTGSNAFRGSAYDSKRGSGYDMQRAP--------- 348
V.vinifera2     ------------YDAPRGPGYDPQRGPGYDAQKGPGYDAQRGPAYDGQRGP--------- 364
R.communis2     ------------YDVSRGSGYDLSRGAAYDAQRGHSYDAQRGPGYNMQRGPSYDAQQRVP 386
A.lyrata        -----------------PGSYDPTIR-----LPTGPYGAPFPPG---------------- 342
A.thaliana      -----------------PGSYDPTTR-----LPTGPYGAPFPPG---------------- 342
P.trichocarpa   ------------GVAQWVGPFDP-SHARG------------------------------- 297
R.communis1     ------------GVAPLGGSCDP-SLAEK------------------------------- 312
V.vinifera1     ------------SGAAWGGAYDPNSHTQH------------------------------- 304
B.distachyon    ---------FDTANAYQPHAAQASAYAGYSGYPVAGYAQ---PSYPGTYAAPQHPV---- 363
HvTGZ21-like    ---------YDSATAYQ---LQANAYASYSGYPVAGYAQ---PNYPGTYAAPQHPV---- 413
O.sativa        ---------YDAATAYQMHAAQANAYAGYPGYPVAGYTQAALPGYPSAYAAPQQPI---- 353
S.bicolor       ---------QAGAYTYAYDAATAYAYAGYSGYP--GYAQSAVPNYS--YAVPPQP----- 356
TGZ21           HISREPRLGHISREPRLGHISRGPSLGHISRGPRLGHISREPRMGHISREPRMGHI---- 456

G.max2          -------------------------------GYD-------------------------- 349
G.max1          -----------------------AYDAPRAAGYDAQSRGVAG-PHGHARPMNNMPYGSTT 384
V.vinifera2     -----------------------VYDAPRGTNYDAPTRAAPG-PHGQVPSGNNVPYGSAT 400
R.communis2     GYDVQRIPGYAPRTPGYDVQQGPHYDASRVAGYDPAARGTAVPPHGQMTPANNVPYGSAT 446
A.lyrata        -------------------------------------------------PSNNTPYGSAA 353
A.thaliana      -------------------------------------------------PSNNTPY---- 349
P.trichocarpa   ------------------------------------------------------------
R.communis1     ------------------------------------------------------------
V.vinifera1     ------------------------------------------------------------
B.distachyon    -----------------------TSGTATDVTCTYGAVGSAGYPAGVVQPSTGAANAGQA 400
HvTGZ21-like    -----------------------ASGAATDATSAYGAAGSTGYPAAPVQASSGAANAGQA 450
O.sativa        -----------------------SSGVATDVASMYGAISSAGYPAGVVQSSSGAANAGQA 390
S.bicolor       -----------------------SSGATTEAASMYGAAGSAGYPTAQVQPSSATANAAQP 393
TGZ21           -----------------------SRVLRLEHTTMLMMLARLMHMQVTLAIQLQATRKVQC 493

G.max2          ------------------------------------------------------------
G.max1          PPARSGGGYEPA-RGVNPARR--------------------------------------- 404
V.vinifera2     PPARSGGGYEAAPRGGNPVRR--------------------------------------- 421
R.communis2     PSTRAVSGYEAQARGGNPVRR--------------------------------------- 467
A.lyrata        ----------TASARGNPSCR--------------------------------------- 364
A.thaliana      -----------AGTHGNPSRR--------------------------------------- 359
P.trichocarpa   ------------------------------------------------------------
R.communis1     ------------------------------------------------------------
V.vinifera1     ------------------------------------------------------------
B.distachyon    PPAP--PAPYPATYDPTRGAQR-------------------------------------- 420
HvTGZ21-like    P-----PAPYPATYDPTKAAQR-------------------------------------- 467
O.sativa        P------ATYPVAYDPTRAGQR-------------------------------------- 406
S.bicolor       PPPPPPAAPYPSTYDQTRGAQR-------------------------------------- 415
TGZ21           PTIPMLHLRSQQAAVQLRTPQEASMGQLVVLDILLGKFSRAVALQMQRKHLLLHHHRQHH 553
```

Figure S5. Multiple sequence alignment of coding sequences of *TGZ15*, *TGZ21* and *Poaceae* proteins. Significant sequence similarity across the whole coding region is maintained, but lack of conserved guanosine in position 747 of the *TGZ15* and *TGZ21* cDNA (marked with black solid arrow) results in a frame shift. It preserves the Gly249 amino acid (encoded by GGA instead of GGG) but changes all the consecutive amino acids and extends the protein length with respect to other Poaceae sequences (see Fig.S4 for comparison).

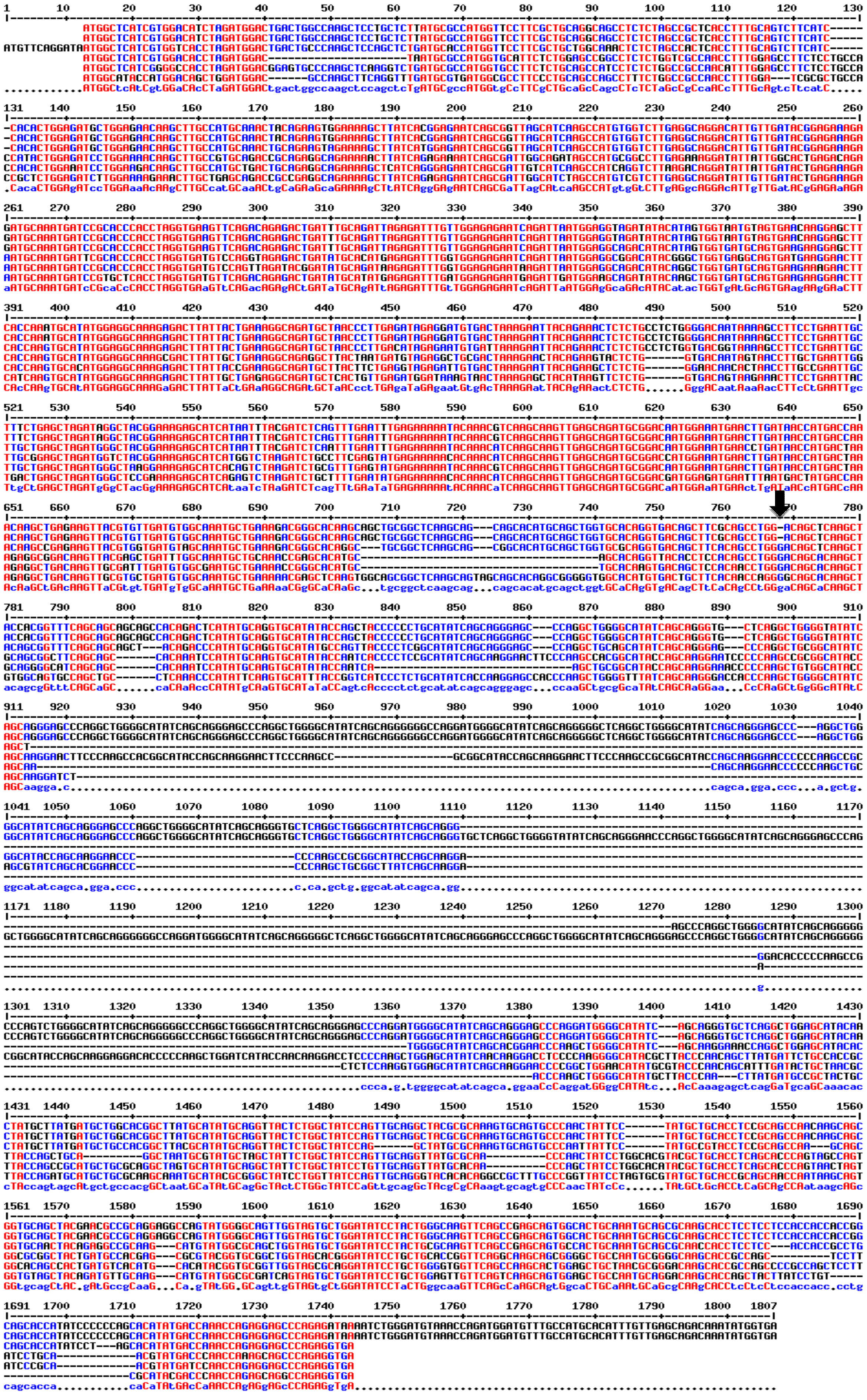

Figure S6. Alignment of *TGZ15* and *TGZ21* 115-nt fragments (704:818) with maize DNA sequences. Sequences were retrieved by blastn search with the query cDNA fragment in the MaizeGDB database. AJ421525 and AJ488103 are *TGZ15* and *TGZ21* database records, respectively. chr2 is genomic DNA of maize chromosome 2.

```
               1       10        20        30        40        50        60        70        80        90        100       110   116
               |--------+---------+---------+---------+---------+---------+---------+---------+---------+---------+---------+-----|
          chr2 CAGCACATGCAGCTGGTGCACAGGTGACAGCTTCGCAGCCTGGGACAGCTCAAGCTACCACGGTTTCAGCAGCAGCAGCCACAGACTCATATGCAGGTGCATATACCAGCTACCCC
MAGIv4.0_43385 CAGCACATGCAGCTGGTGCACAGGTGACAGCTTCGCAGCCTGGGACAGCTCAAGCTACCACGGTTTCAGCAGCAGCAGCCACAGACTCATATGCAGGTGCATATACCAGCTACCCC
    BT055826.1 CAGCACATGCAGCTGGTGCACAGGTGACAGCTTCGCAGCCTGGGACAGCTCAAGCTACCACGGTTTCAGCAGCAGCAGCCACAGACTCATATGCAGGTGCATATACCAGCTACCCC
    AC208515.3 CAGCACATGCAGCTGGTGCACAGGTGACAGCTTCGCAGCCTGGGACAGCTCAAGCTACCACGGTTTCAGCAGCAGCAGCCACAGACTCATATGCAGGTGCATATACCAGCTACCCC
    AC226205.2 CAGCACATGCAGCTGGTGCACAGGTGACAGCTTCGCAGCCTGGGACAGCTCAAGCTACCACGGTTTCAGCAGCAGCAGCCACAGACTCATATGCAGGTGCATATACCAGCTACCCC
 GRMZM2G045005 CAGCACATGCAGCTGGTGCACAGGTGACAGCTTCGCAGCCTGGGACAGCTCAAGCTACCACGGTTTCAGCAGCAGCAGCCACAGACTCATATGCAGGTGCATATACCAGCTACCCC
    AJ421525.2 CAGCACATGCAGCTGGTGCACAGGTGACAGCTTCGCAGCCTGG-ACAGCTCAAGCTACCACGGTTTCAGCAGCAGCAGCCACAGACTCATATGCAGGTGCATATACCAGCTACCCC
    AJ488103.1 CAGCACATGCAGCTGGTGCACAGGTGACAGCTTCGCAGCCTGG-ACAGCTCAAGCTACCACGGTTTCAGCAGCAGCAGCCACAGACTCATATGCAGGTGCATATACCAGCTACCCC
         chr10      CATGCAGCTGGTGCACAGGTGACAGCTTCACAGCCTGGGACAGCTCAAGCTACAGCGGTTCCAGCAGCAGCT---ACAGACCCATATGCAGGTGCATATGCCAGTTACCCC
MAGIv4.0_15607      CATGCAGCTGGTGCACAGGTGACAGCTTCACAGCCTGGGACAGCTCAAGCTACAGCGGTTCCAGCAGCAGCT---ACAGACCCATATGCAGGTGCATATGCCAGTTACCCC
    DY691218.1      CATGCAGCTGGTGCACAGGTGACAGCTTCACAGCCTGGGACAGCTCAAGCTACAGCGGTTCCAGCAGCAGCT---ACAGACCCATATGCAGGTGCATATGCCAGTTACCCC
    DT641696.1      CATGCAGCTGGTGCACAGGTGACAGCTTCACAGCCTGGGACAGCTCAAGCTACAGCGGTTCCAGCAGCAGCT---ACAGACCCATATGCAGGTGCATATGCCAGTTACCCC
    BT063774.1      CATGCAGCTGGTGCACAGGTGACAGCTTCACAGCCTGGGACAGCTCAAGCTACAGCGGTTCCAGCAGCAGCT---ACAGACCCATATGCAGGTGCATATGCCAGTTACCCC
    BT061427.1      CATGCAGCTGGTGCACAGGTGACAGCTTCACAGCCTGGGACAGCTCAAGCTACAGCGGTTCCAGCAGCAGCT---ACAGACCCATATGCAGGTGCATATGCCAGTTACCCC
    EU970708.1      CATGCAGCTGGTGCACAGGTGACAGCTTCACAGCCTGGGACAGCTCAAGCTACAGCGGTTCCAGCAGCAGCT---ACAGACCCATATGCAGGTGCATATGCCAGTTACCCC
    AC217043.3      CATGCAGCTGGTGCACAGGTGACAGCTTCACAGCCTGGGACAGCTCAAGCTACAGCGGTTCCAGCAGCAGCT---ACAGACCCATATGCAGGTGCATATGCCAGTTACCCC
    AC204386.3      CATGCAGCTGGTGCACAGGTGACAGCTTCACAGCCTGGGACAGCTCAAGCTACAGCGGTTCCAGCAGCAGCT---ACAGACCCATATGCAGGTGCATATGCCAGTTACCCC
 GRMZM2G025054      CATGCAGCTGGTGCACAGGTGACAGCTTCACAGCCTGGGACAGCTCAAGCTACAGCGGTTCCAGCAGCAGCT---ACAGACCCATATGCAGGTGCATATGCCAGTTACCCC
    EU975072.1      CATGCAGCTGGTGCACAGGTGACAGCTTCACAGCCTGGGACAGCTCAAGCTACAGCGGTTCCAGCAGCAGCT---ACAGATCCATATGCAGGTGCATATGCCAGTTACCCC
    EU960246.1      CATGCAGCTGGTGCACAGGTGACAGCTTCACAGCCTGGGACAGCTCAAGCTACAGCGGTTCCAGCAGCAGCT---ACAGATCCATATGCAGGTGCATATGCCAGTTACCCC
    CO453662.1                  GCACAGGTGACAGCTTCACAGCCTGGGACAGCTCAAGCTACAGCGGTTCCAGCAGCAGCT---ACAGACCCATATGCAGGTGCATATGCCAGTTACCCC
    DV024912.1                      AGGTGACAGCTTCACAGCCTGGGACAGCTCAAGCTACAGCGGTTCCAGCAGCAGCT---ACAGACCCATATGCAGGTGCATATGCCAGTTACCCC
    DR796649.1                                                      GGACAGCTCAAGCTACAGCGGTTCCAGCAGCAGCT---ACAGACCCATATGCAGGTGCATATGCCAGTTACCCC
    DV172598.1 CAGCACATGCAGCTGGTGCACAGGTGACAGCTTCGCAGCCTGGGACAGCTCAAGCTACCA
     Consensus .....catgcagctggtGCACAGGTGACAGCTTCaCAGCCTGGGACAGCTCAAGCTACagCGGTTcCAGCAGCAGCt...ACAGAccCATATGCAGGTGCATATgCCAGtTACCCC
```

Figure S7. Expression profile of *HvPng1-like* during barley development. A. Comparison of 15 different barley tissue types representing barley developmental stages in two barley genotypes, Morex and Golden Promise. All data were generated in one experiment involving total 63 GeneChip hybridizations (Druka et al. 2006). B. General expression profile during barley development generated by meta-analysis of 1795 GeneChip hybridizations from multiple experiments.

A.

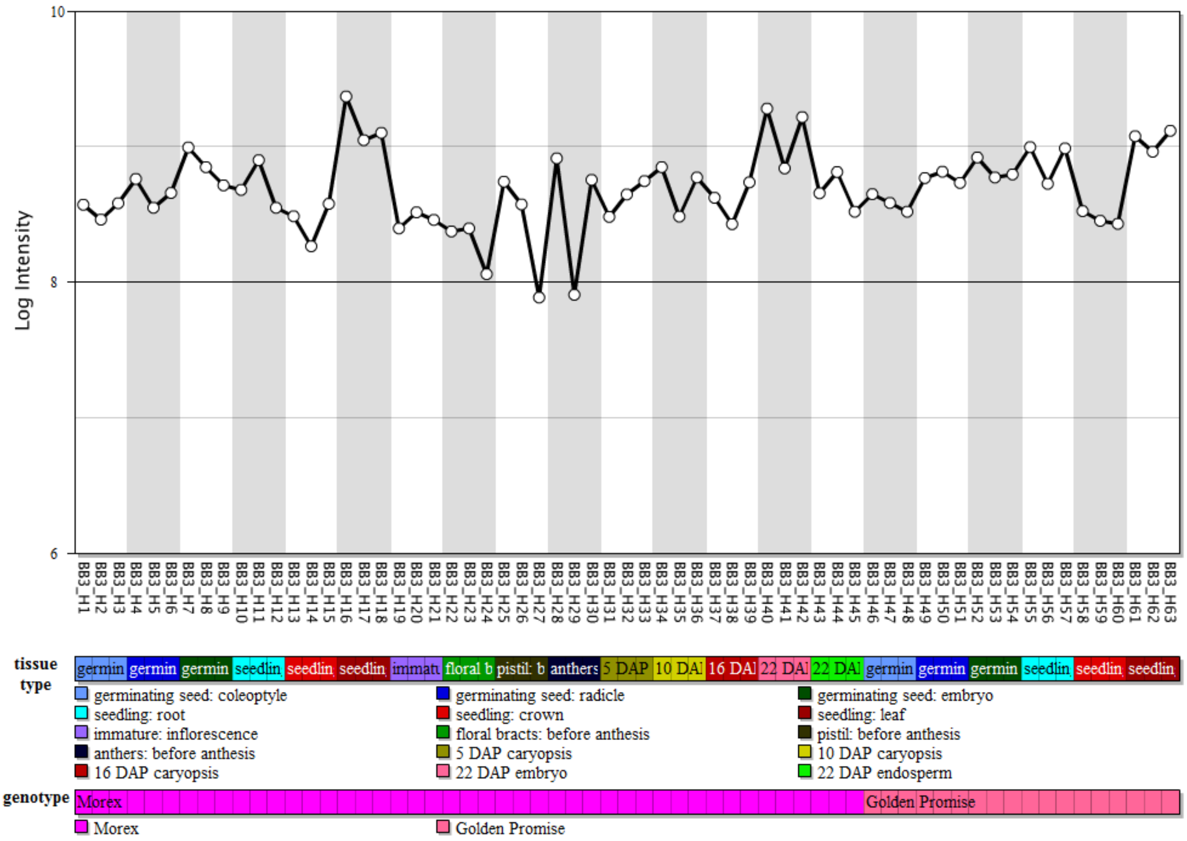

B.

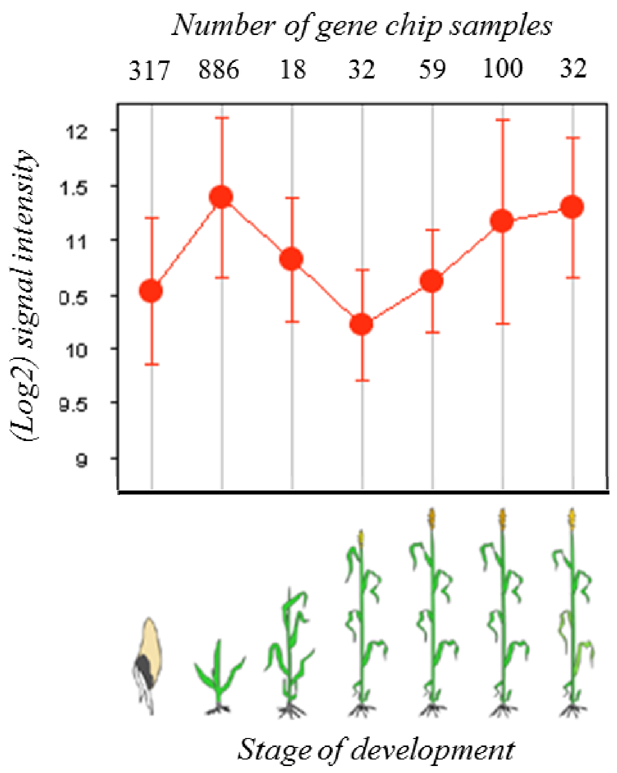

Figure S8. Visual representation of variation for HvPng1-like transcript levels during barley leaves senescence, measured relative to day 0. Data collected from 4 biological replicates, each replicate being a pool of about 30 plants. Whisker-box plots were generated with REST 2009 software.

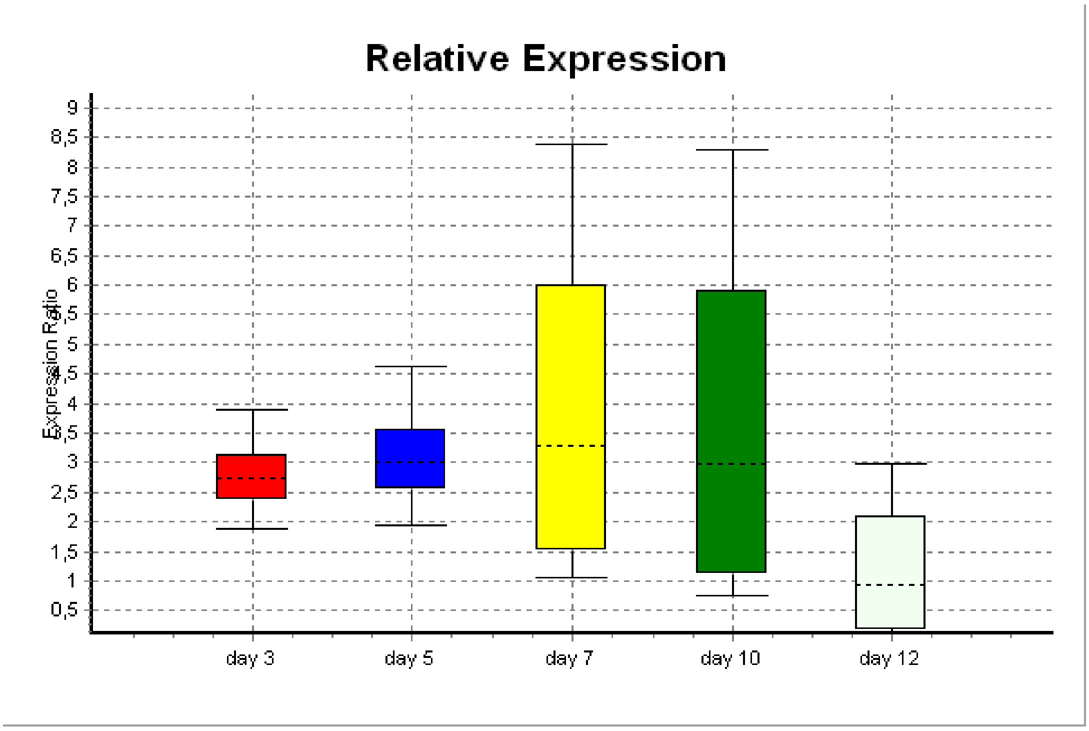

Supplement: Supplementary file 1 — Supplementary material 1 (PDF 444 kb) [file 726_2014_1912_MOESM1_ESM.pdf]
